# Two more pieces of the colibactin genotoxin puzzle from *Escherichia coli* show incorporation of an unusual 1-aminocyclopropanecarboxylic acid moiety†
†Electronic supplementary information (ESI) available: Experimental methods, supplementary tables and figures including information on mutants and NMR spectra of compounds. See DOI: 10.1039/c5sc00101c



**DOI:** 10.1039/c5sc00101c

**Published:** 2015-03-24

**Authors:** Xiaoying Bian, Alberto Plaza, Youming Zhang, Rolf Müller

**Affiliations:** a Department of Microbial Natural Products , Helmholtz-Institute for Pharmaceutical Research Saarland (HIPS) , Helmholtz Centre for Infection Research (HZI) , Department of Pharmaceutical Biotechnology , Saarland University , Campus C2 3 , 66123 Saarbrücken , Germany . Email: rolf.mueller@helmholtz-hzi.de ; Tel: +49-681-30270201; b Shandong University-Helmholtz Institute of Biotechnology , State Key Laboratory of Microbial Technology , School of Life Science , Shandong University , Zhuzhou Road 168 , 266101 Qingdao , P. R. China . Email: zhangyouming@sdu.edu.cn ; Tel: +86-531-88363082

## Abstract

Colibactin represents a structurally undefined class of bacterial genotoxin inducing DNA damage and genomic instability in mammalian cells, thus promoting tumour development and exacerbating lymphopenia in animal models. The colibactin biosynthetic gene cluster (*clb*) has been known for ten years and it encodes a hybrid nonribosomal peptide synthetase (NRPS)/polyketide synthase (PKS) assembly line. Nevertheless, the final chemical product(s) remain unknown. Previously, we and others reported several colibactin pathway-related metabolites including *N*-myristoyl-d-asparagine (**1**) as part of a prodrug precursor that is cleaved from the putative precolibactin to form active colibactin by the peptidase ClbP. Herein, we report two new colibactin pathway-related metabolites (**2** and **3**) isolated from a *clbP* mutant of the probiotic *E. coli* Nissle 1917 strain. Their structures were established by HRMS and NMR. Compound **2** shows an additional 4-aminopenatanoic acid moiety with respect to **1**, while **3** is characterized by the presence of an unusual 7-methyl-4-azaspiro[2.4]hept-6-en-5-one residue. Moreover, we propose the biosynthetic pathway towards both intermediates on the basis of extensive gene inactivation and feeding experiments. The identification of **2** and **3** provides further insight into colibactin biosynthesis including the involvement and formation of a rare 1-aminocyclopropanecarboxylic acid unit. Thus, our work establishes additional steps of the pathway forming the bacterial genotoxin colibactin.

## Abstract 2

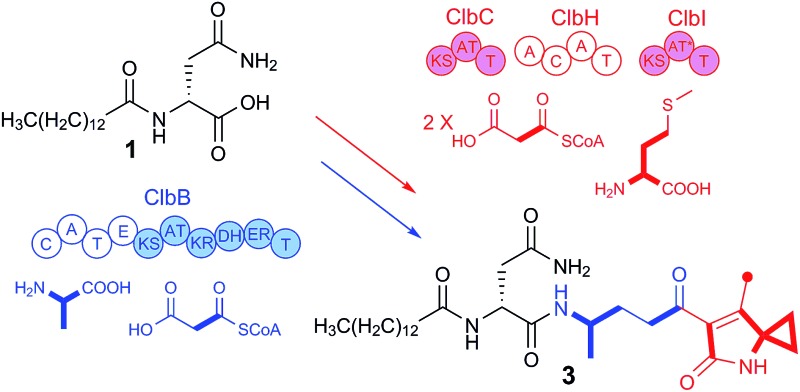
Biosynthetic pathway intermediates related to genotoxin colibactin formation: a linear compound **3** bearing a rare 7-methyl-4-azaspiro[2.4]hept-6-en-5-one residue.

## Introduction

Human gut microbiota play an important role in health and disease.^1,2^ As the predominant aero-anaerobic Gram-negative bacteria in the normal intestinal microbiota, most of the *Escherichia coli* are harmless or of benefit to the mammalian host and rarely cause disease. However, some virulent *E. coli* have acquired pathogenicity islands and can induce disease. Recently, it has been shown that many commensal and extra-intestinal pathogenic *E. coli* strains of the B2 phylogenetic group and closely related *Enterobacteriaceae* harbour a *pks* genomic island that is responsible for the production of a bacterial genotoxin termed colibactin.^3,4^ Colibactin-producing *E. coli* (*clb*+) induce DNA damage, cell cycle arrest, and genomic instability of mammalian cells *in vitro* and *in vivo*, and thus promote development of tumours, *e.g.* colorectal cancer, and exacerbate lymphopenia in animal models.^3,5–11^ To reveal the molecular mechanism underlying the genotoxic activity of colibactin and to design drugs that inhibit its activity, information regarding colibactin's chemical structure, biosynthetic assembly and transfer to the host cell is required.^12^

The colibactin biosynthetic gene cluster (*clb*) was described about a decade ago and encodes a hybrid nonribosomal peptide synthetase (NRPS)/polyketide synthase (PKS) assembly line as reported for many secondary metabolite biosynthetic pathways.^3,13^ However, the product(s) of this pathway remain a mystery which is at least in part due to the fact that the cytopathic activity is contact-dependent and therefore neither the *clb*+ bacterial culture supernatant nor the bacterial lysate are cytopathic.

Although the probiotic commensal strain *E. coli* Nissle 1917 is widely used for the treatment of intestinal disorders, it surprisingly also contains a functional *clb* locus.^14^ Unexpectedly, the product(s) of the colibactin pathogenicity island here possess anti-inflammatory activity and are required for the probiotic activity of *E. coli* Nissle 1917.^15^ A possible explanation for these entirely different activities might be that colibactin may not be a single compound but rather a class of metabolites differentially expressed and formed under various culture conditions. Thereby the *clb* biosynthetic gene cluster may encode for the formation of additional compounds that could mediate the probiotic activity observed in the gut.^15^ These intriguing but ambiguous results motivated our efforts to learn more about the chemical structure and the biosynthesis of colibactin(s).

**Fig. 1:**
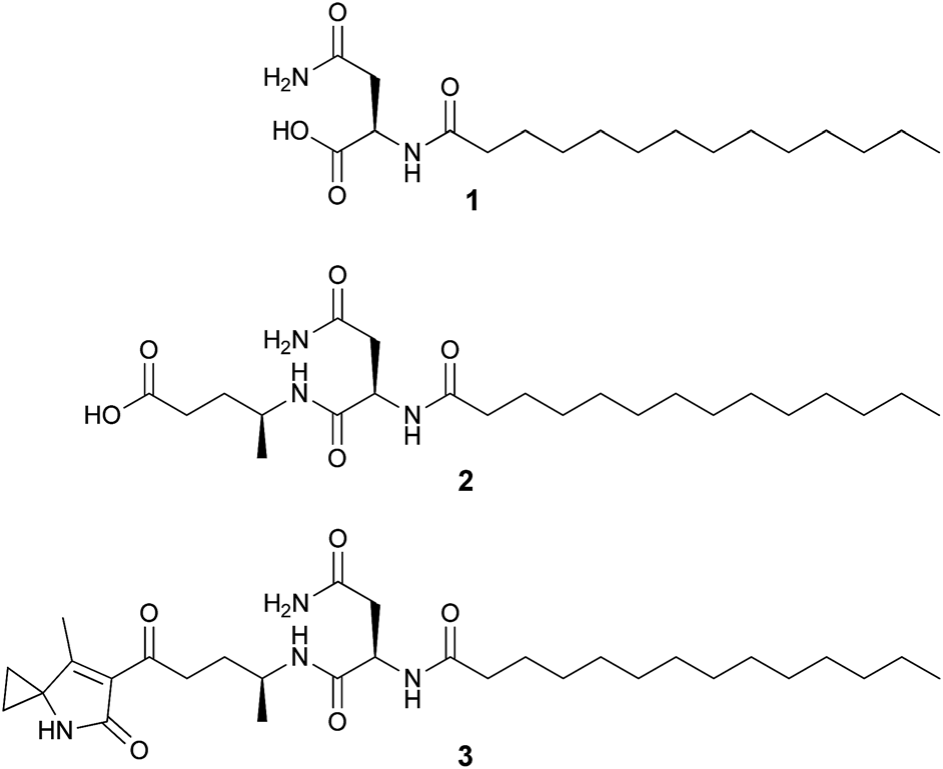
Structures of colibactin pathway-related metabolites **1–3**.

Our previous genetic and comparative metabolomic efforts led to the identification of several colibactin pathway-dependent small molecules including *N*-myristoyl-d-asparagine (**1**, Fig. 1).^16^ Compound **1** is believed to represent a fragment of a prodrug precursor cleaved from the putative precolibactin to form active colibactin by a peptidase ClbP (see Fig. 4Ba).^16–19^ Such a prodrug activation mechanism was described recently in several natural product biosynthetic pathways, *e.g.* xenocoumacins,^20^ zwittermicins,^21,22^ and didemnins.^23^ The *in vitro* biochemical characterization of ClbP and *in vivo* identification of **1** provided evidence for a prodrug mechanism during colibactin maturation.^16,17^ More recently, a handful of colibactin pathway intermediates including **1** and **2** (Fig. 1) were identified from the wild type and/or *clbP* mutants of *E. coli* Nissle 1917 and *E. coli* IHE3034 by comparative metabolomics and targeted structural network analyses.^24^ However, the structure of **2** was only proposed based on MS^2^ fragmentation analysis.

Herein, we identify two new colibactin pathway-related products (**2** and **3**) isolated from the *clbP* mutant of *E. coli* Nissle 1917. Their structures were established by HRMS and NMR and their biosynthetic pathway(s) was proposed by a combination of gene inactivation and feeding experiments revealing further insight into colibactin formation.

## Results and discussion

We applied Red/ET recombineering to construct multiple single or double gene mutations/deletions in the native colibactin producer *E. coli* Nissle 1917.^25,26^ Each inactivation of the respective *clb* biosynthetic gene by replacement with an antibiotic resistance gene was performed as previously reported (see Experimental section, Tables S1 and S2, Fig. S1†).^16^ The gene *clbL* encodes a putative amidase that might catalyse the hydrolysis of an amide in the putative precolibactin,^3,24^ whereas *clbP* encodes a peptidase which cleaves **1** off from precolibactin.^16–19^ Thus, *clbL* or *clbP* single deletion mutants were constructed to investigate the presence of colibactin-related products. Mutants and wild type strains were cultured, extracted, and analysed by LC-MS. We observed that the yield of the precursor fragment *N*-myristoyl-d-asparagine (**1**) is greatly decreased in the *clbP* mutant but slightly increased in the *clbL* single mutant (Fig. S2†). These results were consistent with our previous reports.^16^

Intriguingly, the LC-MS chromatograms of the *clbP* mutant showed additional metabolites when compared to the wild type and the *clbL* mutant (Fig. S2†). To shed light on their molecular structures, EtOAc extracts prepared from 20 L cultures of *E. coli* Nissle 1917 Δ*clbP* were chromatographed by sephadex LH-20 followed by reversed-phase HPLC to yield compounds **2** (4.9 mg) and **3** (0.7 mg).

The molecular formula of compound **2** was determined to be C_23_H_43_N_3_O_5_ by HR-ESI-MS (*m*/*z* 442.3273 [M + H]^+^). Its structure was characterized by 2D NMR and HRMS experiments and it exhibits an additional 4-aminopentanoic acid (App) residue in comparison to **1** (Fig. 1, NMR data see Table S3, Fig. S3–S6†). The structure of **2** was first proposed by Vizcaino *et al.* on the basis of MS fragmentation analysis but the compound was not isolated or characterized by NMR.^24^ Our structural elucidation of **2** supports the hypothesis that the NRPS module (C–A–T, Fig. 4Ba) found on ClbB activates alanine and incorporates this residue into an *N*-myristoyl-d-asparagine precursor (**1**) as formed by ClbN (Fig. 4Ba). This process is followed by extension of the intermediate by the PKS module (KS–AT–KR–DH–ER–T) on ClbB by one malonyl-CoA unit including β-carbon processing (Fig. 4Ba), and subsequent hydrolytic release of **2** by a currently unknown mechanism. This hydrolysis pattern is also described in other colibactin pathway intermediates.^24^

**Fig. 2:**
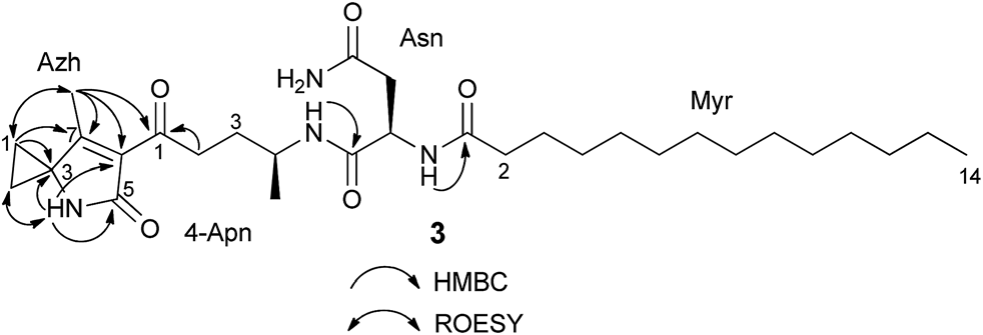
NMR-based connectivity of the fragments of **3**.

HR-ESI-MS of **3** displayed an [M + H]^+^ ion peak at *m*/*z* 547.3853 consistent with the molecular formula C_30_H_50_N_4_O_5_. A mass consistent with **3** was detected by MS before,^24^ but it was never isolated nor structurally characterized. Its ^1^H NMR spectrum in comparison to that of **2** showed a few additional signals including an exchangeable NH proton at *δ* 8.53 (1H, s) and a methyl vinyl at *δ* 1.97 (3H, s) (see Table S4†). Comparison of the HSQC spectrum of **3** to that of **2** indicated that the signals corresponding to the methylene C-2_4-Apn_ (*δ*_H_ 2.14, 2.17, *δ*_C_ 30.4) in **2** were shifted downfield in **3** (*δ*_H_ 2.92, 2.83; *δ*_C_ 38.5). Additionally, analysis of ^13^C NMR, HSQC, and DET 135 spectra of **3** indicated the presence of two equivalent methylene groups (*δ*_H_ 1.49, *δ*_C_ 13.6, and *δ*_H_ 1.41, *δ*_C_ 13.7) that were absent in **2**. A detailed analysis of the 2D NMR data (HSQC, HMBC, DQF-COSY, ROESY) of **3** showed that the C-terminus of the fragment *N*-myristoyl-asparagine was connected to a 4-aminopentanoyl (4-Apn) residue. The structure of the remaining C_7_H_8_NO fragment was assembled as follows: HMBC correlations from the methyl signal at *δ* 1.97 (Me-7_Azh_) to the quaternary carbon resonance at *δ* 45.3 (C-3_Azh_) and to the olefinic carbon resonances at *δ* 169.5 (C-7_Azh_) and 128.9 (C-6_Azh_), together with HMBC correlations from the amide proton at *δ* 8.53 (NH-4_Azh_) to C-3_Azh_, C-6_Azh_, and to the carbonyl resonance at *δ* 169.2 (C-5_Azh_), determined the presence of a 4-methyl-1,5-dihydro-2*H*-pyrrol-2-one ring (see Fig. 2). Furthermore, long-range correlations from the methylene protons at *δ* 1.49 (H-1_Azh_) and 1.41 (H-2_Azh_) to C-3_Azh_ and C-7_Azh_, COSY correlations between H-1_Azh_ and H-2_Azh_, together with ROESY correlations from H-1_Azh_ to Me-7_Azh_ and from H-2_Azh_ to H-4_Azh_ indicated that a cyclopropane was spiro fused to the dihydropyrrolone ring forming a 7-methyl-4-azaspiro[2.4]hept-6-en-5-one residue (Azh). Finally, connectivity between Azh and 4-Apn residues was deduced from four-bond HMBC correlations from Me-7_Azh_ and NH-4_Azh_ to the ketone carbon at *δ* 197.7 (C-1_4-Apn_) thereby completing the structure of **3** as depicted in Fig. 1 (NMR spectra see Fig. S7–S15†). Tandem mass spectrometry provided further evidence to support the structure of **3** (Fig. S16†). MS^2^ fragmentation of the ion peak at *m*/*z* 547 displayed ions at *m*/*z* 530 [M + H–NH_3_]^+^, 320 [M + H–NH_2_–Myr]^+^, and 205 [M + H–Myr–Asn–NH_2_]^+^. Thus the MS^2^ fragmentation patterns in complete agreement with the structure of **3** determined by NMR.

The absolute configuration of d-Asn was determined by MS detected chromatographic comparison with appropriate amino acid standards of the acid hydrolysate of **2** and **3** after derivatizing with l- and d-FDLA (l-fluoro-2,4-dinitro-phenyl-5-l/d-leucinamide).^27,28^ Similarly, the absolute configuration of C-4 of App in **2** was established as *S*. The *S* configuration of C-4_Apn_ in **3** was assumed as *S* on the basis the structural and NMR data similarities to those of **2**.

**Fig. 3:**
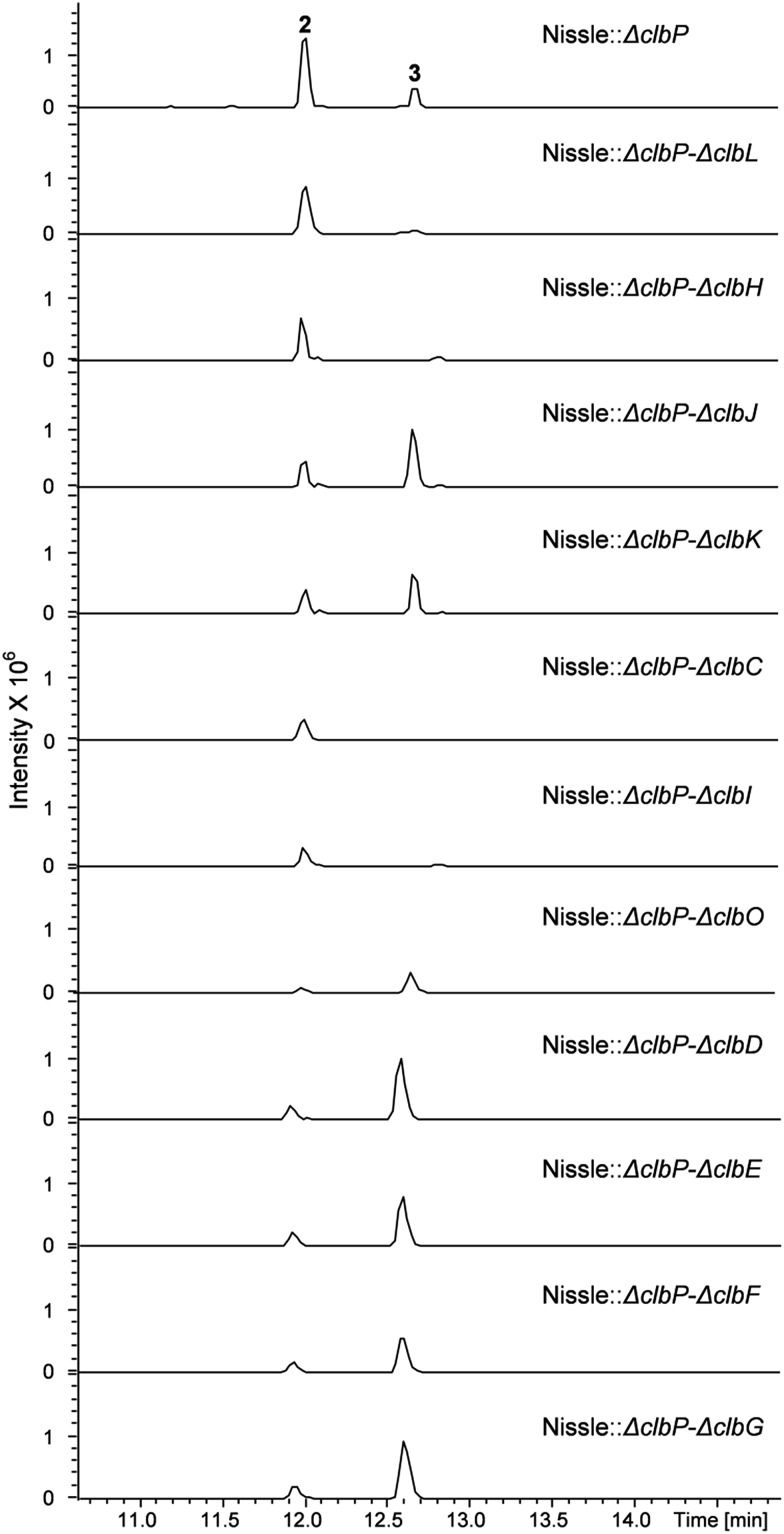
UPLC-HRMS analysis of the *E. coli* Nissle double mutants. Shown is a base peak chromatogram (BPC) *m*/*z* for 442.3 [M + H]^+^ and 547.4 [M + H]^+^ of compounds **2** and **3**, respectively.

To investigate the question which gene(s) from the *clb* pathway are responsible for biosynthesis of the 7-methyl-4-azaspiro[2.4]hept-6-en-5-one moiety in **3**, eleven mutations were performed on the *clbP* mutant of *E. coli* Nissle 1917 by one more step of Red/ET recombination. Each target gene was replaced by a kanamycin resistance gene (*Km* ^*R*^) for *clbC* to *clbK* and *clbO*, whereas inactivation of *clbL* was performed by replacement with a spectinomycin resistance gene (*Spect* ^*R*^) (Table S1†). All mutants were verified by colony PCR (data now shown). LC-MS analyses of the *clbP* mutant and the double mutants showed that inactivation of *clbC*, *clbH* or *clbI* resulted in abolishment of the production of **3** indicating that these three genes were involved in the biosynthesis of **3** (Fig. 3). On the other hand, gene inactivation of each of *clbD*, *clbE*, *clbF*, *clbG*, *clbJ*, *clbK* or *clbO* had no significant effect on the production of **3** (Fig. 3). The *clbL*/*clbP* double mutant showed a great decrease of **3** implying that the putative peptide amidase ClbL might be involved in hydrolysis of **3** from a larger precolibactin molecule. Unfortunately, we were not able to detect any larger colibactin-pathway dependent metabolites in these mutants.

**Fig. 4:**
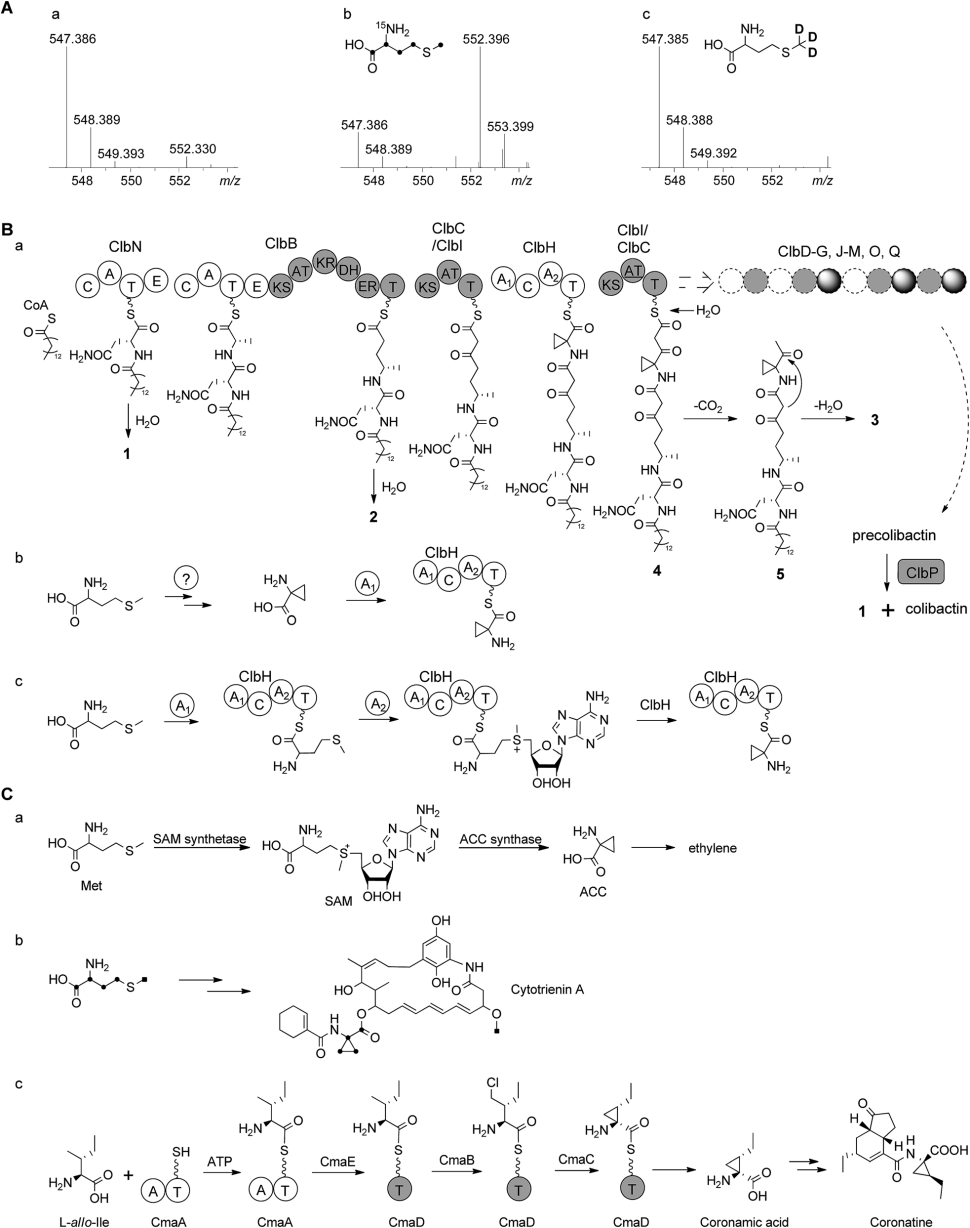
(A) HR-ESI-MS spectra of **3** obtained from feeding experiments. (a) Control; (b) after feeding of l-[U-^13^C, ^15^N]methionine (only four carbons and one nitrogen are found in the final product, Fig. S17†); (c) after feeding of l-[methyl-^2^H_3_]methionine (no d incorporated) showing that the aminobutyryl moiety of methionine (without *S*-methyl) is incorporated into the final product **3**. (B) (a) Proposed biosynthetic pathway of colibactin up to intermediate **4** involving **1** and **2** tethered to the assembly line. After release of **4** and decarboxylation of the β-keto-acid, **3** is formed by intramolecular cyclization analogous to Dieckmann condensation. (b) A hypothesis for ACC biosynthesis and then loading on ClbH; (c) alternative hypothesis for loading of methionine onto ClbH and then conversion to ACC on the T domain catalyzed by A_2_ domain. (C) Biosynthesis of ACC moiety in other organisms. (a) Biosynthesis of ACC in the ethylene pathway in higher plants;^30,31^ (b) incorporation of l-[U-^13^C]methionine into cytotrienin A showed that the ACC unit originates from methionine in *Streptomyces* sp. RK95-74;^33^ (c) biosynthetic pathway of the coronamic acid (CMA) moiety of coronatine in *P. syringae* strains.^34,35^ C, condensation domain; A, adenylation domain; T, thiolation domain; E, epimerization domain; KS, ketosynthase domain; AT, acyltransferase domain; DH, dehydratase domain; KR, ketoreductase domain; ER, enoyl reductase domain; SAM, *S*-adenosylmethionine; ACC, 1-aminocyclopropanecarboxylic acid.

According to the gene inactivation experiments and bioinformatics analysis of the modular organization of the pathway, a proposal for the biosynthesis of **3** is shown in Fig. 4B. Initially the presence of the 7-methyl-4-azaspiro[2.4]hept-6-en-5-one residue in **3** seemed difficult to rationalize biosynthetically. However, when taking into account the possibility of incorporation of a nonproteinogenic 1-aminocyclopropane-carboxylic acid (ACC) residue in conjunction with hydrolysis and possible shunt product formation the following scenario was developed: compound **2** undergoes extension by one PKS module (encoded by ClbC or ClbI; KS–AT–T) to incorporate one malonyl-CoA unit, one NRPS module (ClbH, A_1_–C–A_2_–T) is needed for incorporation of the unusual ACC, and one more PKS module (encoded by ClbI or ClbC; KS–AT–T) is needed to incorporate an additional malonyl-CoA unit eventually giving rise to **4** attached to the biosynthetic assembly line (Fig. 4Ba). We hypothesize that **4** might be hydrolysed enzymatically (*e.g.* TE catalysed, like **2**) to form the corresponding free acid undergoing decarboxylation to build **5**, which in turn can engage in an intramolecular cyclization analogous to a Dieckmann condensation forming the dihydropyrrolone ring found in **3** (Fig. 4Ba). A similar Dieckmann cyclization catalyzed by a Dieckmann cyclization domain (DKC) was proposed to form the pyrrolone ring in pyranonigrin E,^29^ but no homologous DKC domain was found in the *clb* gene cluster leading to the speculation that the condensation in **3** may occur spontaneously.

The ACC moiety is quite an unusual moiety in bacterial secondary metabolites, and to the best of our knowledge this is the first occurrence in an *E. coli* natural product. However, it is well known from plant metabolites such as in the biosynthesis of the higher plant hormone ethylene where ACC serves as key intermediate,^30^ ACC is synthesized from *S*-adenosylmethionine (SAM) *via* the pyridoxal phosphate (PLP)-dependent ACC synthase catalysing a γ-elimination reaction (Fig. 4Ca).^30,31^ The ansa-bridged cytotrienin from the soil bacterium *Streptomyces* sp. RK95-74 also contains an ACC moiety^32^ and stable isotope feeding experiments showed that the ACC moiety in this producer is also derived from l-methionine (Fig. 4Cb),^33^ but the cytotrienin biosynthetic gene cluster has not been published yet. An alternative strategy was described for the enzymatic formation of the ethyl-substituted ACC moiety coronamic acid (CMA) in the phytotoxin coronatine produced by *Pseudomonas syringae* strains. Here, biosynthesis follows a cryptic chlorination pathway relying on an aminoacyl thioester intermediate involving five dedicated proteins CmaABCDE (Fig. 4Cc).^34^ CmaB and CmaC execute γ-halogenation of l-*allo*-isoleucine followed by intramolecular γ-elimination to form the cyclopropyl ring.^34,35^ As no homologues of ACC synthase genes or coronamic acid biosynthesis genes were found in the colibactin gene cluster, we performed feeding experiments to investigate the hypothetical biosynthesis of ACC in **3**. Methionine labelled by stable isotopes was added to the growth medium resulting in the following incorporation patterns in **3**: feeding of l-[U-^13^C, ^15^N]methionine gave *m*/*z* 552.3959 [M + H]^+^ (5 amu more than 547.3853) corresponding to the chemical formula of **3**-^13^C_4_, ^15^N (C_26_^13^C_4_H_50_N_3_^15^NO_5_, *m*/*z* 552.3959 [M + H]^+^) while feeding of l-[methyl-^2^H_3_]methionine did not result in deuteration of **3** (Fig. 4A, Fig. S17A†). Taken together, these data indicated that the aminobutyryl moiety (accounting for four carbons and one nitrogen without the *S*-methyl carbon) of l-methionine was introduced into the ACC unit of **3** which suggests that ACC originates from l-methionine in *E. coli* (Fig. 4AB, Fig. S17B†). Nonetheless, the l-methionine-ACC pathway including ACC synthase has to the best of our knowledge not been detected in prokaryotic microorganisms.^33,36^ As stated above we did not find a homolog of the ACC synthase gene in the *E. coli* Nissle 1917 *clb* gene cluster. An additional search for homologs in the genome of *E. coli* Nissle 1917 (GenBank: CP007799.1) using ACC synthases from apple (*Malus domestica*) (GenBank: AAB68617.1, PDB: ; 1B8G)^37^ and from *Penicillium citrinum* (GenBank: AB038512)^38^ as queries was performed using BlastP.^39^ This resulted in the identification of some homologs belonging to the family of PLP-dependent aminotransferases/transaminases/lyases exhibiting low identities on the amino acid level (<30%, Table S5†). As either one of these proteins or any other ORF of unknown function encoded in the *E. coli* genome may be responsible for the conversion of SAM to ACC, clearly further *in vivo* gene inactivation and *in vitro* biochemical experiments are required to study the formation of ACC in *E. coli*.

Intriguingly, the NRPS module encoded on ClbH hypothesized to be in charge of ACC incorporation contains two A domains; the first A domain (A_1_) is predicted to activate a serine residue according to the specificity-conferring code of adenylation domains in NRPSs (Table S6†).^40,41^ A subsequent feeding experiment employing l-[2,3,3-^2^H_3_]serine led to an isotopic pattern compatible with incomplete incorporation of serine into **2** and **3** (Fig. S18A†). This finding may be explained by serine degradation *via* pyruvate to acetyl-CoA and malonyl-CoA for incorporation into **2** and **3** by the PKS or fatty acid synthetase (FAS) which may be responsible for the fatty acid side chain (Fig. S18B†). Thus, we assume that the A_1_ domain is not required for serine activation but may play a role in ACC formation as discussed below. The second A domain (A_2_) of ClbH is predicted to be specific for a hydrophobic/aromatic residue (phenyalanine/tryptophan/tyrosine/valine/leucine) or may represent an A domain with novel specificity (Table S6†). Feeding of l-[U-^2^H]valine, l-[U-^2^H]leucine, l-[U-^13^C, ^15^N]phenyalanine and l-[ring-^2^H_4_]tyrosine did not show any incorporation into **2** or **3** (data not shown). The nonribosomal code of the A_2_ domain of ClbH showed that the strictly conserved aspartic acid (D235) interacting with the α-amino group of amino acids in peptide synthetases is mutated to alanine (Table S6†), indicating that the substrate of the A_2_ domain may not have an α-amino group.^42^ Thus, according to the chemical structure of **3**, we speculate that the A_1_ domain may load an ACC unit (Fig. 4Bb) or methionine (Fig. 4Bc) onto ClbH. The former hypothesis regarding loading of ACC by the A_1_ domain would lead to the requirement of ACC biosynthesis in *E. coli* and make the A_2_ domain ‘superfluous’ (Fig. 4Bb). The latter hypothesis postulates that ClbH might function as a SAM synthetase after methionine loading by the A_1_ domain to the T domain of ClbH. Subsequently, ClbH may also function as an ACC synthase (Fig. 4Bc). According to this hypothesis the SAM synthetase and ACC synthase activity may be encoded within the NRPS ClbH. In light of this hypothesis the biosynthetic function of each domain found on ClbH (A_1_–C–A_2_–T) during loading, modification and ACC incorporation needs to be characterized in upcoming experiments in detail. To achieve this goal the whole protein and/or the respective domains need to be expressed in their active forms, but this is a goal for future experiments.

We measured the *in vitro* bacterial growth inhibitory activity of **2** and **3**, as **1** has been reported to exhibit mild growth inhibitory activity against *Bacillus subtilis* NCIB 3610.^24^ Both, **2** and **3**, also showed weak growth inhibitory activities (EC_50_ 30–70 μg mL^–1^) against *E. coli* (TolC-deficient) and *B. subtilis* DSM-10 (Fig. S19†).

According to our gene inactivation experiments, further assembly of precolibactin and colibactin most likely requires additional proteins encoded on the *clb* pathogenicity island. Whereas this study provides evidence on the function of ClbC, ClbH and ClbI *in vivo* on route to intermediate **4**, it does not determine any activity for ClbDEFG, ClbJ, ClbK and ClbO which are clearly not required for the formation of **3** according to the mutagenesis experiments reported here. Consequently, further experiments *in vitro* and/or *in vivo* are required not only to determine the structure of colibactin and precolibactin but also to decipher each biosynthetic step in greater detail.

## Conclusions

Colibactin represents a chemically unknown class of bacterial genotoxin present in most species of the B2 group of *E. coli*. Its presence in the probiotic *E. coli* Nissle 1917 not only contributes to probiotic activity but also induces chromosomal instability of mammalian cells. With the aim to eventually determine the structure of colibactin, two colibactin pathway-related metabolites were identified by HRMS and NMR analyses from *E. coli* Nissle 1917 after extensive mutagenesis studies. Compound **3** is a linear lipopeptide containing a novel 7-methyl-4-azaspiro[2.4]hept-6-en-5-one (Azh) residue and its biosynthetic pathway was proposed based on gene inactivation, *in silico* analysis of biosynthetic pathway proteins and feeding experiments. Surprisingly, the latter indicated that the uncommon nonproteinogenic amino acid ACC, which is involved in the formation of Azh, might be derived from l-methionine in *E. coli* and possible scenarios for its biosynthesis during colibactin assembly are discussed. The structural elucidation and the biosynthetic proposal regarding these two colibactin pathway-dependent biosynthetic intermediates (**2** and **3**) provides additional knowledge required on route to eventually establish the structure of colibactin and thus deepens our understanding of this intriguing probiotic and genotoxic compound class.
