# Supplementary material for: Two more pieces of the colibactin genotoxin puzzle from Escherichia coli show incorporation of an unusual 1-aminocyclopropanecarboxylic acid moiety
Source: Chem Sci. 2015 Mar 24;6(5):3154–60. doi: 10.1039/c5sc00101c (PMC5490422; doi:10.1039/c5sc00101c)
Supplement: Supplementary file 1 [file SC-006-C5SC00101C-s001.pdf]

Electronic Supplementary Material (ESI) for Chemical Science.
This journal is © The Royal Society of Chemistry 2015

Supplementary Information

Two more pieces of the colibactin genotoxin puzzle from *Escherichia coli* show incorporation of an unusual 1-aminocyclopropanecarboxylic acid moiety

Xiaoying Bian,[a,b] Alberto Plaza,[a] Youming Zhang,[b,*] and Rolf Müller[a,*]

[a]Department of Microbial Natural Products, Helmholtz-Institute for Pharmaceutical Research Saarland (HIPS), Helmholtz Centre for Infection Research (HZI) and Department of Pharmaceutical Biotechnology, Saarland University, Campus C2 3, 66123 Saarbrücken, Germany.

[b]Shandong University-Helmholtz Institute of Biotechnology, State Key Laboratory of Microbial Technology, Shandong University, Zhuzhou Road 168, 266101 Qingdao, P. R. China.

* To whom correspondence should be addressed:

RM: Tel: +49 681 30270201; Fax: +49 681 30270202; E-mail: rolf.mueller@helmholtz-hzi.de

YZ: Tel: +86 531 88363082; Fax: +86 531 88363203; E-mail: zhangyouming@sdu.edu.cn

Table of Contents

1. Experimental section

1.1 Gene inactivation by replacement of an antibiotic resistance marker in the chromosome of *E. coli* Nissle 1917 using Red/ET recombineering

1.2 General methods for cultivation and culture extraction for LC-MS analysis

1.4 Isolation of compounds 2 and 3

1.4 LC-MS Analysis of L/D-FDLA Derivatives

1.5 Feeding experiments

1.6 Antibacterial activity test

2. Supplementary Tables

Table S1. List of *E. coli* Nissle 1917 mutants constructed in this study

Table S2. Primers used in this study

Table S3. NMR Spectroscopic Data for Compound **2** (DMSO-d_6)

Table S4. NMR Spectroscopic Data for Compound **3** (DMSO-d_6)

Table S5 Possible homologous genes of ACC synthase in *E. coli* Nissle 1917

Table S6 Analysis of the Specificity-Conferring Residues in residues in ClbH

3. Supplementary Figures

Fig. S1 An example diagram for *clb* gene inactivation (*clbH* in this figure) in the chromosome of *E. coli* Nissle 1917

Fig. S2 UPLC-HRMS analysis (BPC 340-550 + All MS) of the *E. coli* Nissle 1917 wild type and mutants.

Fig. S3 ^{1}H NMR spectrum of **2** in DMSO-d_6

Fig. S4 HSQC spectrum of **2** in DMSO-d_6

Fig. S5 HMBC spectrum of **2** in DMSO-d_6

Fig. S6 DQF-COSY spectrum of **2** in DMSO-d_6

Fig. S7 ^{1}H NMR spectrum of **3** in DMSO-d_6

Fig. S8 ^{13}C NMR spectrum of **3** in DMSO-d_6

Fig. S9 ^{13}C NMR (DEP-135) spectrum of **3** in DMSO-d_6

Fig. S10 HSQC spectrum of **3** in DMSO-d_6

Fig. S11 HMBC spectrum of **3** in DMSO-d_6

Fig. S12 Selective HMBC spectrum of **3** in DMSO-d_6

Fig. S13 DQF-COSY spectrum of **3** in DMSO-d_6

Fig. S14 2D-HOHAHA spectrum of **3** in DMSO-d_6

Fig. S15 2D-ROESY spectrum of **3** in DMSO-d_6

Fig. S16 HR-MS/MS Spectrum (maXis) of compound **3**

Fig. S17 HRMS analysis of feeding of L-methionine-$^{13}C_5$,^{15}N into *E. coli* Nissle 1917 mutant

Fig. S18 Feeding of L-serine-d_3 to *E. coli* Nissle 1917 mutant led to multiply deuterated peaks of compounds **2** (A) and **3** (B)

Fig. S19 Growth inhibition of compounds **2** and **3** against *E. coli* (TolC-deficient) and *B. subtillis* DSM-10

4. Supplementary References

1. Experimental section

1.1 Gene inactivation by replacement of an antibiotic resistance marker in the chromosome of *E. coli* Nissle 1917 using Red/ET recombineering

The target genes were replaced by an antibiotic selection marker (chloramphenicol resistance gene *Cm*^{R}, kanamycin resistance gene *Km*^{R}, or spectinomycin resistance gene *Spect*^{R}) using Red/ET recombineering. The detailed procedure of Red/ET recombineering is provided in previous publications.[1-4] The antibiotic resistance genes flanked with homology arms (~50 bp) were generated by polymerase chain reaction (PCR) amplification using Phusion high-fidelity polymerases (Thermo-Scientific) according to the manufacture's manual, and the templates for *Cm*^{R}, *Km*^{R} and *Spect*^{R} were plasmids Pirate-cm-new (GeneBridges), Pirate-km (GeneBridges), and pR6K-Spect-BAC (GeneBridges), respectively. For Red/ET recombineering, purified PCR products of resistance genes flanked by different homology arms, were transformed into *E. coli* Nissle 1917 containing the recombinase expression plasmid pSC101-BAD-gbaA-tet by electroporation, respectively.[1;5] Recombinants were selected on LB plates containing 20 μg/mL chloramphenicol, 20 μg/mL kanamycin, or 40 μg/mL spectinomycin, respectively. To generate the double mutants, the antibiotic resistance gene was used to replace the target gene by Red/ET recombineering in *E. coli* Nissle 1917 *ΔclbP* containing pSC101-BAD-gbaA-tet. Correct clones were verified by colony PCR (Table S1). The recombinase expression plasmid pSC101-BAD-gbaA-tet was removed by culture temperature shift to 42 ℃. A list of mutants generated in this study is provided as Table S1. Oligonucleotides used for gene deletions are listed in Table S2. An exemplarily example of a diagram for gene inactivation (*clbH*) by replacement of an antibiotic resistance marker in the chromosome of *E. coli* Nissle 1917 using Red/ET recombineering is shown in Fig. S1.

1.2 General methods for cultivation and culture extraction for LC-MS analysis

The *E. coli* strains Nissle 1917 wildtype and verified mutants were incubated in 25 mL Luria broth in 100 mL glass flasks amended with suitable antibiotics at 30℃ (200 rpm) for 2 days. The culture was extracted by 25 mL EtOAc. Twenty mL organic phase was evaporated to dryness. The resulting residue was

dissolved in 200 μL of methanol for LC-MS analysis. Standard analysis of crude extracts was performed on a Dionex Ultimate 3000 LC system using a Waters Acquity BEH C-18, 50 x 2 mm, 1.7 μm dp column. Separation of a 2 μL sample was achieved by a linear gradient with (A) H_2O + 0.1 % formic acid (FA) to (B) acetonitrile (ACN) + 0.1 % FA at a flow rate of 600 μL/min and at 45 ℃. The gradient was initiated by a 0.5 min isocratic step at 5 % B, followed by an increase to 95 % B in 9 min to end up with a 1.5 min step at 95 % B before re-equilibration employing initial conditions. UV spectra were recorded by a DAD in the range from 200 to 600 nm. The MS measurement was carried on an amaZon speed mass spectrometer (BrukerDaltonics, Bremen, Germany) using the standard ESI source. Mass spectra were acquired in centroid mode ranging from 200 – 2000 *m/z* in positive ionization mode with auto MS2 fragmentations. Ultra Performance Liquid Chromatography coupled to High Resolution Mass Spectrometry (UPLC-HRMS) measurements were performed on a Dionex Ultimate 3000 RSLC system using a Waters BEH C18, 50 x 2.1 mm, 1.7 μm dp column. Separation of 2 μL sample was achieved by a linear gradient with (A) H_2O + 0.1 % FA to (B) ACN + 0.1 % FA at a flow rate of 600 μL/min and 45 ℃. The gradient was initiated by a 1 min isocratic step at 5 % B, followed by an increase to 95 % B in 6 min to end up with a 1.5 min step at 95 % B before reequilibration with initial conditions. UV spectra were recorded by a DAD in the range from 200 to 600 nm. The LC flow was split to 75 μL/min before entering the maXis4Ghr-ToF mass spectrometer (BrukerDaltonics, Bremen, Germany) using the standard ESI source. Mass spectra were acquired in centroid mode ranging from 150 – 2000 *m/z* at 2 Hz scan speed.

1.3 Isolation of compounds 2 and 3

E. coli Nissle 1917 *ΔclbP* was cultured in 10 x 5 L-flask containing 2 L LB medium for 2 days at 30 ℃, 150 rpm. Thirty mL preautoclaved XAD-16 adsorber resin (Sigma) was added into each flask after 24 h incubation. The resin (from 20 L culture) was separated from the supernatant by sieving, washed with deionized H_2O (1 L x 3), and then extracted with EtOAc (2.0 L x 3) to get 2.15 g residue. The EtOAc extract was partitioned with MeOH: Hexane (1:1) to obtain a dried 1.76 g MeOH extract which was further fractionated through a Sephadex LH-20 column using MeOH as the mobile phase. Sephadex

fractions 34-39 were chromatographed on semi-preparative HPLC using a RP-C12 column (Jupiter 4μ Proteo 90A, 250 ×100 mm, 4 micron). The mobile phase consisted of H_2O (A) containing 0.05% formic acid (FA) and acetonitrile (ACN) (B). The gradient used was as follows: 0-3min 30%B, 3-8min 30%-65%B, 8-22min 65-76%B. 22-22.5min 76-95%B. 4.9 mg of **2** (t_R 19.4 min) and 0.7 mg of **3** (t_R 21.3min) were obtained.

Compound 2: colorless amorphous solid, $[\alpha]^{20}_{D}$ + 23.3 (*c* 0.2, MeOH), IR (film) ν_{max} 3681, 2940, 2856, 2076, 1739, 1455, 1365, 1228, 1055, 1013 cm^{-1}, ^1H and ^{13}C NMR data see Table S1, HR-ESI-MS *m/z* 442.3273 $[M+H]^+$ (calcd for $C_{23}H_{44}N_3O_5$, 442.3281)

Compound 3: colorless amorphous solid, $[\alpha]^{20}_{D}$ + 13.4 (*c* 0.1, MeOH), IR (film) ν_{max} 3707, 2922, 2826, 2075, 1738, 1455, 1371, 1228, 1055, 1013 cm^{-1}, ^1H and ^{13}C NMR data see Table S2, HR-ESI-MS *m/z* 547.3853 $[M+H]^+$ (calcd for $C_{30}H_{51}N_4O_5$, 547.3859)

1.4 LC-MS Analysis of L/D-FDLA Derivatives

Approximately 0.25 mg of compound was hydrolyzed with 6 N HCl (0.5 mL) for 16 h at 100 ℃, dried, and dissolved in H_2O (100 μL). To a 50 μL aliquot of each were added 1 N $NaHCO_3$ (20 μL) and 1% 1-fluoro-2,4-dinitrophenyl-5-L-leucinamide (L-FDLA or D-FDLA solutions in acetone, 100 μL),[6;7] and the mixtures were heated to 40 ℃ for 40 min, allowed to cool to room temperature, neutralized with 2 N HCl (20 μL), and evaporated to dryness. Residues were dissolved in ACN and analyzed by LC-MS. All measurements were performed on a Dionex Ultimate 3000 RSLC system using a Waters BEH C18, 100 x 2.1 mm, 1.7 μm column by injection of 1 μL sample. Separation was achieved by a gradient using (A) H_2O + 0.1% FA to (B) ACN + 0.1% FA at a flow rate of 550 μL/min and 45 ℃. The gradient was as follows: starting at 5 % B to increase to 10 % B in 1 min, from 1 to 15 min increase to 35 % B, from 15 to 22 min increase to 50% B, from 22 to 25 min increase to 80 % B. After a 1 min hold at 80 % B the system was reequilibrated with initial conditions for 5 minutes. UV data was acquired at 340 nm and MS detection was performed simultaneously. Coupling the HPLC to the MS was supported by an Advion

Triversa Nanomate nano-ESI system attached to a Thermo Fisher Orbitrap. LC flow is split to 500 nL/min before entering the ion source. Mass spectra were acquired in centroid mode ranging from 150 – 1000 *m/z* at a resolution of R = 30000.

Retention times (t_R, min) of the FDLA-derivatized amino acids for compounds **2** and **3**: L-Asn 13.4, D-Asn 14.3 *m/z* 428 $[M+H]^+$ (analyzed as Asp), 4*R*-App 19.2, 4*S*-App 17.6 *m/z* 412 $[M+H]^+$.

1.5 Feeding experiments

E. coli Nissle 1917 *ΔclbP/ΔclbJ* double mutant was cultivated in Luria-Bertani (LB) broth supplemented with 20 μg/mL kanamycin and 20 μg/mL chloramphenicol. L-valine-d_8 (Deutero), L-leucine-d_{10} (Deutero), L-Serine-d_3 (Cambridge Isotope Laboratories), L-phenylalanine $^{13}C_9$,^{15}N (Aldrich), L-Tyrosine-phenyl-d_4 (Sigma-Aldrich), L-Methionine-$^{13}C_5$,^{15}N (Campro Scientific) and L-methionine-*methyl*-d_3 (Sigma-Aldrich) (final concentration 0.5 mg/mL) were dissolved into 5 mL LB medium supplemented with suitable antibiotics and sterilized by syringe filter (0.2 μm), respectively. The medium was inoculated with 2% overnight culture of *E. coli* Nissle 1917 *ΔclbP/ΔclbJ* and cultivated at 30 ℃ for 2 days; the culture was extracted by the same volume of EtOAc, the organic layer was dried by Genevac EZ-2 plus, and then redisolved into 50 μL MeOH for LC-MS measurement. Standard analysis of crude extracts was performed on a Dionex Ultimate 3000 LC system using a Waters Acquity BEH C-18, 50 x 2 mm, 1.7 μm dp column. Separation of a 2 μL sample was achieved by a linear gradient with (A) H2O + 0.1 % FA to (B) ACN + 0.1 % FA at a flow rate of 0.6 mL/min and 45 ℃. The gradient was initiated by a 0.5 min isocratic step at 5 % B, followed by an increase to 95 % B in 9 min to end up with a 1.5 min step at 95 % B before reequilibration with initial conditions. UV spectra were recorded by a DAD in the range from 200 to 600 nm. The MS measurement was carried on an amaZon speed mass spectrometer (BrukerDaltonics, Bremen, Germany) using the standard ESI source. Mass spectra were acquired in centroid mode ranging from 200 – 2000 *m/z* in positive ionization mode with auto MS2 fragmentations. UPLC-HRMS was done on Accela UPLC-system (Thermo-Fisher) coupled to a linear trap-FT-Orbitrap

combination (LTQ-Orbitrap) in positive ionization mode with a Waters BEH RP-C18 column (50 x 2 mm; 1.7 μm particle diameter, flow rate 0.6 mL/min) with a mobile phase of H_2O/ACN each containing 0.1% of FA, using a gradient from 5–95% of ACN over 9 min.

1.6 Antibacterial activity test

The activity measurement was same to the description on references.[8;9] The DMSO stocks of compounds **2** and **3** were serially diluted in Müller Hinton broth. OD_{600} was measured after 4, 8, 16 h of treatment and the starting OD was 0.01, which corresponds to approximate 5 x 10^6 cfu/mL. Two bacteria *Bacillus subtilis* DSM-10 (DSMZ) and *E. coli* (TolC-deficient) (Internal Strain Collection) were tested in this study.

2. Supplementary Tables

Table S1. List of *E. coli* Nissle 1917 mutants constructed in this study

Name	Description and Construction	Primers (Forward/Reverse) sequences see Table S2	Ref.
E. coli Nissle 1917	wild type		Ardeypharm, Germany
E. coli Nissle 1917 *Δclb*	*Δclb-cm*, deletion of *clb* gene cluster, the *clbA-clbQ* region was completely replaced by a chloramphenicol resistance gene (*Cm*^{R}) by Red/ET recombineering in *E. coli* Nissle 1917 containing a recombinase expression plasmid pSC101-BAD-gbaA-tet	For Red/ET: clb-cm-new5/clb-cm-new3<br>For colony PCR: clb-ch1/clb-ch2; clb-ch3/clb-ch4	This study
E. coli Nissle 1917 *ΔclbP*	*ΔclbP-cm*, deletion of *clbP* gene, the *clbP* gene was replaced by a chloramphenicol resistance gene (*Cm*^{R}) by Red/ET recombineering in *E. coli* Nissle 1917 containing a recombinase expression plasmid pSC101-BAD-gbaA-tet	For Red/ET: clbP-cm5/ clbP-cm3<br>For colony PCR: clbL-ch5/clbP-ch3	This study
E. coli Nissle 1917 *ΔclbL*	*ΔclbL-cm*, deletion of *clbL* gene, the *clbL* gene was replaced by a chloramphenicol resistance gene (*Cm*^{R}) by Red/ET recombineering in *E. coli* Nissle 1917 containing a recombinase expression plasmid pSC101-BAD-gbaA-tet	For Red/ET: clbL-cm5/ clbL-cm3<br>For colony PCR: clbL-ch5/clbL-ch3	This study
E. coli Nissle 1917 *ΔclbP /ΔclbL*	*ΔclbP-cm & ΔclbL-spect*, deletion of *clbL* in *E. coli* Nissle 1917 *ΔclbP*, the *clbL* gene was replaced by a spectionmyicin resistance gene (*Spect*^{R}) by Red/ET recombineering in *E. coli* Nissle 1917 *ΔclbP*.	For Red/ET: clbL-spect5/ clbL-spect3<br>For colony PCR: clbL-ch5/clbL-ch3	This study
E. coli Nissle 1917 *ΔclbP /ΔclbH*	*ΔclbP-cm & ΔclbH-km*, deletion of *clbH* in *E. coli* Nissle 1917 *ΔclbP*, the *clbH* gene was replaced by a kanamycin resistance gene (*Km*^{R}) by Red/ET recombineering in *E. coli* Nissle 1917 *ΔclbP* containing a recombinase expression plasmid pSC101-BAD-gbaA-tet (Fig. S1)	For Red/ET: clbH-km5/ clbH-km3<br>For colony PCR: clbH-ch1/km-ch1; km-ch2/clbH-ch2	This study
E. coli Nissle 1917 *ΔclbP /ΔclbJ*	*ΔclbP-cm & ΔclbJ-km*, deletion of *clbJ* in *E. coli* Nissle 1917 *ΔclbP*, the *clbJ* gene was replaced by a kanamycin resistance gene (*Km*^{R}) by Red/ET recombineering in *E. coli* Nissle 1917 *ΔclbP* containing a recombinase expression plasmid pSC101-BAD-gbaA-tet	For Red/ET: clbJ-km5/ clbJ-km3<br>For colony PCR: clbJ-ch1/km-ch1; km-ch2/clbJ-ch2	This study
E. coli Nissle 1917 *ΔclbP*	*ΔclbP-cm & ΔclbK-km*, deletion of *clbK* in *E. coli* Nissle 1917 *ΔclbP*, the *clbK* gene was	For Red/ET: clbK-km5/ clbK-km3	This study

/*ΔclbK*	replaced by a kanamycin resistance gene (*Km^R*) by Red/ET recombineering in *E. coli* Nissle 1917 *ΔclbP* containing a recombinase expression plasmid pSC101-BAD-gbaA-tet	For colony PCR: clbK-ch1/km-ch1; km-ch2/clbK-ch2	
E. coli Nissle 1917 *ΔclbP* /*ΔclbC*	*ΔclbP-cm & ΔclbC-km*, deletion of *clbC* in *E. coli* Nissle 1917 *ΔclbP*, the *clbC* gene was replaced by a kanamycin resistance gene (*Km^R*) by Red/ET recombineering in *E. coli* Nissle 1917 *ΔclbP* containing a recombinase expression plasmid pSC101-BAD-gbaA-tet	For Red/ET: clbC-km5/ clbC-km3 For colony PCR: clbC-ch1/km-ch1; km-ch2/clbC-ch2	This study
E. coli Nissle 1917 *ΔclbP* /*ΔclbI*	*ΔclbP-cm & ΔclbI-km*, deletion of *clbI* in *E. coli* Nissle 1917 *ΔclbP*, the *clbI* gene was replaced by a kanamycin resistance gene (*Km^R*) by Red/ET recombineering in *E. coli* Nissle 1917 *ΔclbP* containing a recombinase expression plasmid pSC101-BAD-gbaA-tet	For Red/ET: clbI-km5/ clbI-km3 For colony PCR: clbI-ch1/km-ch1; km-ch2/clbI-ch2	This study
E. coli Nissle 1917 *ΔclbP* /*ΔclbO*	*ΔclbP-cm & ΔclbO-km*, deletion of *clbO* in *E. coli* Nissle 1917 *ΔclbP*, the *clbO* gene was replaced by a kanamycin resistance gene (*Km^R*) by Red/ET recombineering in *E. coli* Nissle 1917 *ΔclbP* containing a recombinase expression plasmid pSC101-BAD-gbaA-tet	For Red/ET: clbO-km5/ clbO-km3 For colony PCR: clbO-ch1/km-ch1; km-ch2/clbO-ch2	This study
E. coli Nissle 1917 *ΔclbP* /*ΔclbD*	*ΔclbP-cm & ΔclbD-km*, deletion of *clbD* in *E. coli* Nissle 1917 *ΔclbP*, the *clbD* gene was replaced by a kanamycin resistance gene (*Km^R*) by Red/ET recombineering in *E. coli* Nissle 1917 *ΔclbP* containing a recombinase expression plasmid pSC101-BAD-gbaA-tet	For Red/ET: clbD-km5/ clbD-km3 For colony PCR: clbD-ch1/km-ch1; km-ch2/clbD-ch2	This study
E. coli Nissle 1917 *ΔclbP* /*ΔclbE*	*ΔclbP-cm & ΔclbE-km*, deletion of *clbE* in *E. coli* Nissle 1917 *ΔclbP*, the *clbE* gene was replaced by a kanamycin resistance gene (*Km^R*) by Red/ET recombineering in *E. coli* Nissle 1917 *ΔclbP* containing a recombinase expression plasmid pSC101-BAD-gbaA-tet	For Red/ET: clbE-km5/ clbE-km3 For colony PCR: clbE-ch1/km-ch1; km-ch2/clbE-ch2	This study
E. coli Nissle 1917 *ΔclbP* /*ΔclbF*	*ΔclbP-cm & ΔclbF-km*, deletion of *clbF* in *E. coli* Nissle 1917 *ΔclbP*, the *clbF* gene was replaced by a kanamycin resistance gene (*Km^R*) by Red/ET recombineering in *E. coli* Nissle 1917 *ΔclbP* containing a recombinase expression plasmid pSC101-BAD-gbaA-tet.	For Red/ET: clbF-km5/ clbF-km3 For colony PCR: clbF-ch1/km-ch1; km-ch2/clbF-ch2	This study
E. coli Nissle 1917 *ΔclbP*	*ΔclbP-cm & ΔclbG-km*, deletion of *clbG* in *E. coli* Nissle 1917 *ΔclbP*, the *clbK* gene was	For Red/ET: clbG-km5/ clbG-km3	This study

/ΔclbG	replaced by a kanamycin resistance gene (*Km^R*) by Red/ET recombineering in *E. coli* Nissle 1917 *ΔclbP* containing a recombinase expression plasmid pSC101-BAD-gbaA-tet	For colony PCR: clbG-ch1/km-ch1; km-ch2/clbG-ch2

Table S2. Primers used in this study

primer	Sequence (5’→3’)
clb-cm-new5	CGGAATATGAAAATCAATATTATCGACGGCTCAGAAGTGTCTAGATTATCCGTGGCGT GTGACGGAAGATCACTTCGCAG
clb-cm-new3	TGATGATGGAACAGCCATATCTATTGCTCCTTGTATAGTTACACAACTATTACGCCCCG CCCTGCCACTCATC
clb-ch1	TGCCGCCGTATTTGGACTTCCAG
clb-ch2	TCAGTGGGGAGGCAAACGGTAAGC
clb-ch3	TGCTATTAGTTCTCACTGCGAGC
clb-ch4	TGTATTTCAGGCCAAGGCGCGCAC
clbL-spect5	TATCGTAGCGCAGGGACACTGTTGGCACAGTTGGCGTCCGGAGAAAACGTGACTGGC AAGAGATA
clbL-spect3	CGCACCTTTATTGGCGCTGTCACCGATATCCGCCTCTAGTACCCTTATAATTTTTTTAA TCTGTT
clbL-ch5	TCGGGCAGGAGATTCCCATTG
clbL-ch3	TGATGACCATGATCTTACCCGC
clbP-cm5	TGACAATAATGGAACACGTTAGCATTAAAACATTATATCATCTCCTGTGCTGTATACC TGTGACGGAAGATCACTTC
clbP-cm3	TAGAATTCGTTTAATTTGATGATTTAATGTCAGAACGAAAGCTAACAGGATAATTACG CCCCGCCCTGCCACTC
clbP-ch5	TGGAGAAAACCTGGCGTTCATTG
clbP-ch3	TCAGCGACGGCATCCACCATC
clbH-km5	TATGGAACAGCAAGGGATTATGAGACAGTTGCCTACCGACGACCAAACGCTCGAGCT ACTGGGCTATCTG
clbH-km3	TCAGTGATGACTGTCGGTTGTGGCTGCCCAGCGCGTGTGACCAAAGGCCATCAGAAGA ACTCGTCAAGAAG
km-ch1	TCATAGCCGAATAGCCTCTCC
km-ch2	TGCCTGCTTGCCGAATATCATG
clbH-ch1	TGGTGCTGGGGGATAACATG
clbH-ch2	TAGCCCGCATCTTCGAAGGCTG
clbJ-km5	TCATGACGATACATCATGCCGCATTGGCGCGAATGTTACCGGCGGAACTCGAGCTACT GGGCTATCTGGAC
clbJ-km3	TAACGCTGACGCTGGTGACGACGTACAAGTCGTTGCTGGGCGCGGGATCGTCAGAAG AACTCGTCAAGAAGG
clbJ-ch1	TGTCATCCCTAGCGAACCGTC
clbJ-ch2	TGGTACTCACGCCCGCATATAG
clbK-km5	TGACTTACAGTGAAAGCGATATTGCCATTGTTGGCATGAACTGCCGGTACTCGAGCTA CTGGGCTATCTGGAC
clbK-km3	TACGCCGCAACGCCAGATTCTCGCGCGCAGCGCTGACCTGCGAAACCTCATCAGAAG AACTCGTCAAGAAGG
clbK-ch1	TCTACATCACAGAAGTCGAGGG
clbK-ch2	TAAGCGTGCGATGGAAGGAG
clbC-km5	TATGGAATACGCAAGCGAAATGAACGGCATGGAAATCGCCATTATTGGTACTCGAGC TACTGGGCTATCTG
clbC-km3	TACTGACAGGCTATTTCGAGGTTGTCTTCGCCCCCCAGTTGCGCCGCGATCAGAAGAA CTCGTCAAGAAGG
clbC-ch1	TCCTGGAAGAGTATCTGGGTC
clbC-ch2	TCGACGCGCCGCAGATCGTATTC
clbI-km5	TGGCAGAGAATGATTTTGGTATAGCTATCATTGGGATGGCGGGGCGTTTCCTCGAGCT

	ACTGGGCTATCTGG
clbI-km3	TCATTAATCATGTCGTTAACTAGCACGGCAAGTGCGGACCCTCCATCATCAGAAGAACTCGTCAAGAAGG
clbI-ch1	TCTTCGATCTCGGTGGACACTC
clbI-ch2	TACCGCATCTAGCGCTGAAGTTG
clbO-km5	TGGCAAAGGATGATTTTACCTGTGGCTCACTGGATATTGCCATTATTGGCCTCGAGCTACTGGGCTATCTGG
clbO-km3	TACCATCAACACATTCGGAGATAAATTCACTGTGCTCACGGATAGCTCAGAAGAACTCGTCAAGAAGG
clbO-ch1	TGGCAAAACGCACGCATGGTG
clbO-ch2	TGATATCATCCCCTTGCCGGAG
clbD-km5	TCAATGATGAACGTGGCGGTAATAGGTGCAGGAGTAATGGGAACTGGCGTCGAGCTACTGGGCTATCTG
clbD-km3	TCACTGCTCGGCGTACGTCTGTTGATAGCGATAAAACCCCTGCCCTGATCAGAAGAACTCGTCAAGAAGG
clbD-ch1	TCAGCATATCCCTTCGCACAC
clbD-ch2	TGGCAAACAGGACTGCCTCTTG
clbE-km5	TCAACAGACGTACGCCGAGCAGTGATTCTGGGTTAACCAGATAGGGATCTCGAGCTACTGGGCTATCTG
clbE-km3	TGCCTCTTGCTGAGCCAGCTCATAATTTTCAGTGCACATAGTTCAGAAGAACTCGTCAAGAAGG
clbE-ch1	TGGTGTACATACTGCAGAGCG
clbE-ch2	TGCCGATATTCGGCTCTGTGAG
clbF-km5	TATGTGCACTGAAAATTATGAGCTGGCTCAGCAAGAGGCAGTCCTCTCGAGCTACTGGGCTATCTG
clbF-km3	TGCTTCACTCCCCATCATGTAATTCATCGCAATTTGGATTTCATGAATTCAGAAGAACTCGTCAAGAAGG
clbF-ch1	TCGATGCAGGGTATTTGGGCGTG
clbF-ch2	TGCTTGGGAAAAGCTCAGCGCAC
clbG-km5	TGACGAAGGATGTCGCACTGATGTTCCCTGGCTCCGGTTCGCAATCTCGAGCTACTGGGCTATCTG
clbG-km3	TACGCCTGTCCGCCGTTGTCGGAAGCCAACCGCGCGTGGCAGGCGTCTCAGAAGAACTCGTCAAGAAGG
clbG-ch1	TGCCACAATGCTGACCCAAAC
clbG-ch2	TGTACGCCAGCGGTAAATAGG

Sequence of homology arms for recombineering are underlined.

Table S3. NMR Spectroscopic Data for Compound **2** (DMSO-d_6)

C	δ_C[a]	δ_H[b] (J in Hz)	HMBC[c]
		**Myr**	
1	172.4		
2	35.8	2.08 t (7.1)	1, 3, 4
3	25.2	1.46 m	1, 2, 4
4-11	28.8	1.24 m	
12	31.3	1.25	13, 14
13	22.2	1.26	12, 14
14	13.8	0.85 t (6.7)	12, 13
		**Asn**	
1	170.2		
2	49.7	4.46 dt (7.9, 6.1)	1, 3, 4
3a	37.3	2.43 dd (15.1, 6.1)	1, 2, 4
3b		2.32 dd (15.1, 7.9)	
4	171.6		
NH		7.91 d (8.1)	2, 3, 1_{Myr}
NH2		7.25, br s	4
		6.84, br s	3
		**4-App**	
1	174.1		
2	30.4	2.17, 2.14 m	1, 3, 4
3	30.8	1.59 m	1, 2, 4
4	43.8	3.72 m	
5	20.4	0.99 d (6.7)	3, 4
NH		7.49 d (8.4)	4, 1_{Asn}

[a]Recorded at 125 MHz; referenced to residual DMSO-d_6 at δ 39.51 ppm.
[b]Recorded at 500 MHz; referenced to residual DMSO-d_6 at δ 2.50 ppm.
[c]Proton showing HMBC correlation to indicated carbon.

Table S4. NMR Spectroscopic Data for Compound **3** (DMSO-d_6)

C	δ_C[a]	δ_H[b] (J in Hz)	HMBC[c]
		**Myr**	
1	172.1		
2	35.1	2.08 t (7.1)	1, 3, 4
3	25.2	1.45 m	1, 2, 4
4-11	28.8	1.23 m	
12	31.2	1.25	13, 14
13	22.2	1.25	12, 14
14	13.9	0.86 t (6.7)	12, 13
		**Asn**	
1	170.3		
2	49.8	4.48 dt (7.9, 6.1)	1, 3, 4
3a	37.5	2.46 dd (15.1, 6.1)	1, 2, 4
3b		2.31 dd (15.1, 7.9)	
4	171.4		
NH		7.87 d (8.1)	2, 3, 1_{Myr}
NH2		7.23, br s	4
		6.81, br s	3, 4
		**4-Apn**	
1	197.7		
2	38.5	2.92 ddd (15.6, 8.5, 6.8)	1, 3, 4
		2.83 ddd (14.7, 8.5, 6.0)	1, 3, 4
3	29.9	1.60 m	1, 2, 4, 5
4	43.8	3.72 m	2, 3, 5, 1_{Asn}
5	20.5	1.01 d (6.7)	3, 4
NH		7.47 d (8.1)	3, 4, 5, 1_{Asn}
		**Azh**	
1	13.6	1.49 m	2, 3, 7
2	13.7	1.41 m	1, 3, 7
3	45.3		
NH-4		8.53 s	3, 5, 6, 7, $1_{4\text{-}Apn}$
5	169.2		
6	128.9		
7	169.5		
Me-7	10.7	1.97 s	3, 6, 7, $1_{4\text{-}Apn}$

[a]Recorded at 125 MHz; referenced to residual DMSO-d_6 at δ 39.51 ppm.
[b]Recorded at 500 MHz; referenced to residual DMSO-d_6 at δ 2.50 ppm.
[c]Proton showing HMBC correlation to indicated carbon.

Table S5 Possible homologous genes of ACC synthase in *E. coli* Nissle 1917

Accession	Protein(aa)	function	Identities	Positives	E-value
**Query: ACC synthase from *Penicillium citrinum* (GenBank: AB038512) 431aa**					
CCQ05778.1	391	Cystathionine beta-lyase	80/354(23%)	140/354(39%)	1e-12
CCQ08881.1	412	Uncharacterized PLP-dependent aminotransferase YfdZ	85/357(24%)	147/357(41%)	2e-10
CCQ08803.1	405	Alanine transaminase	76/369(21%)	142/369(38%)	6e-09
CCQ07346.1	387	Aspartate aminotransferase	43/158(27%)	70/158(44%)	2e-08
CCQ05105.1	390	Cystathionine beta-lyase	82/350(23%)	142/350(40%)	6e-08
CCQ07384.1	468	hypothetical protein	47/170(28%)	75/170(44%)	9e-05
**Query: ACC synthase *Malus domestica* (GenBank: AAB68617.1) 473aa**					
CCQ07384.1	468	hypothetical protein	79/312(25%)	139/312(44%)	2e-13
CCQ08881.1	412	Uncharacterized PLP-dependent aminotransferase YfdZ	69/313(22%)	133/313(42%)	2e-12
CCQ07608.1	386	Methionine aminotransferase, PLP-dependent	85/347(24%)	145/347(41%)	5e-09
CCQ07883.1	470	Transcriptional regulator, GntR family domain / Aspartate aminotransferase	53/207(26%)	95/207(45%)	2e-08
CCQ05778.1	391	Cystathionine beta-lyase	73/353(21%)	133/353(37%)	5e-08
CCQ08803.1	405	Alanine transaminase	61/318(19%)	132/318(41%)	6e-07
**Query: ACC synthase *Solanum lycopersicum* (GenBank: NP_001234178) 485aa**					
CCQ08881.1	412	Uncharacterized PLP-dependent aminotransferase YfdZ	85/366(23%)	162/366(44%)	5e-20
CCQ08803.1	405	Alanine transaminase	87/376(23%)	165/376(43%)	5e-18
CCQ07384.1	468	hypothetical protein	82/330(25%)	144/330(43%)	5e-14
CCQ07883.1	470	Transcriptional regulator, GntR family domain / Aspartate aminotransferase	71/289(25%)	121/289(41%)	1e-10
CCQ05778.1	391	Cystathionine beta-lyase	55/221(25%)	92/221(41%)	1e-07

Table S6 Analysis of the Specificity-Conferring Residues in residues in ClbH

Domain	Nonribosomal Code	Predicted AAs Incorporated		
		PKS/NRPS[10]	NRPSpredictor2[11]	NPRSsp[12]
ClbH-A1	DVWHFSLIDK	Ser	hydrophobic-aliphatic<br>Ser	Ser
ClbH-A2	**A**SGSLGGICK	No hit<br>novel	hydrophobic-aromatic<br>phe/trp/phg/tyr/bht/val	Phe

3. Supplementary Figures

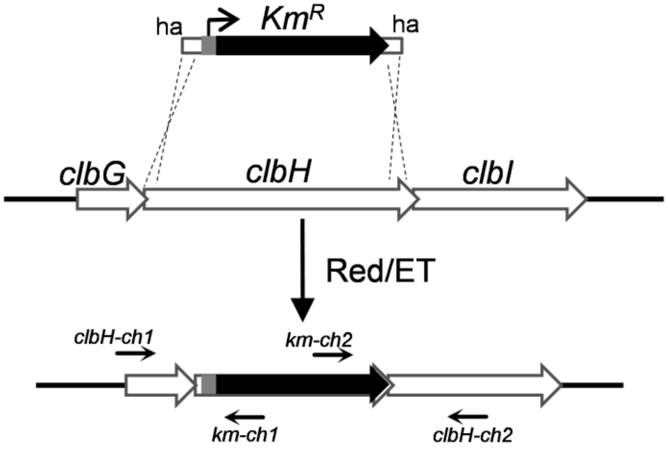

Fig. S1 An example diagram for *clb* gene inactivation (*clbH* in this figure) in the chromosome of *E. coli* Nissle 1917. The colony PCR using two pairs of primers (clbH-ch1 and km-ch1) and (km-ch2-clbH-ch2) was used for verification of correct mutants (Tables S1 and S2). ha: homology arm; Km^R: kanamycin resistance gene; Red/ET: Red/ET recombineering.

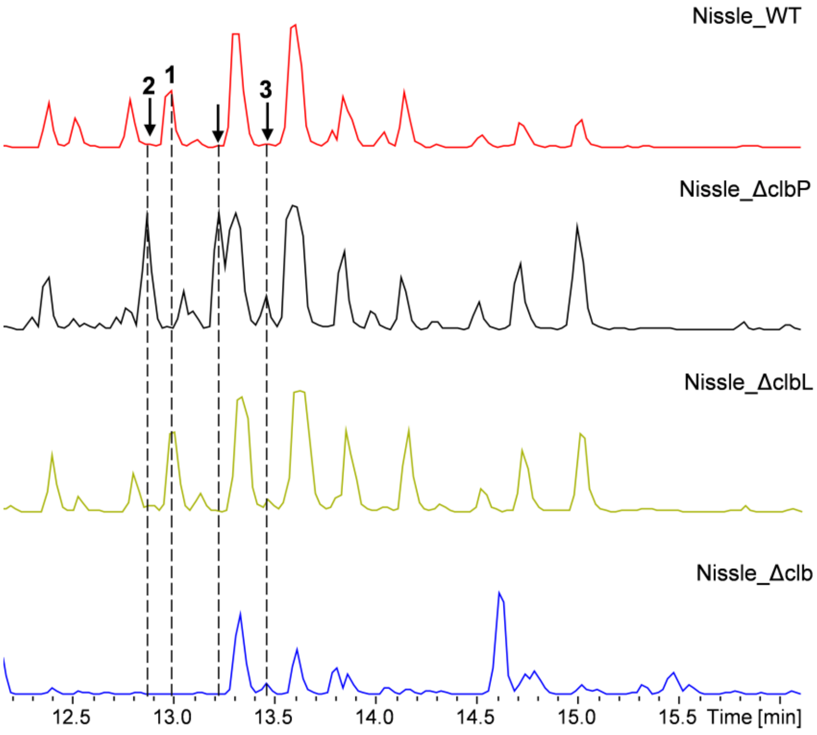

Fig. S2 UPLC-HRMS analysis (BPC 340-550 + All MS) of the *E. coli* Nissle 1917 wild type and mutants. The indicated peaks including **2** and **3** represent the compounds present in the *clbP* mutant but absent in WT, *clb* mutant and *clbL* mutant. The arrow without a number represents an ion *m/z* 456.3430.

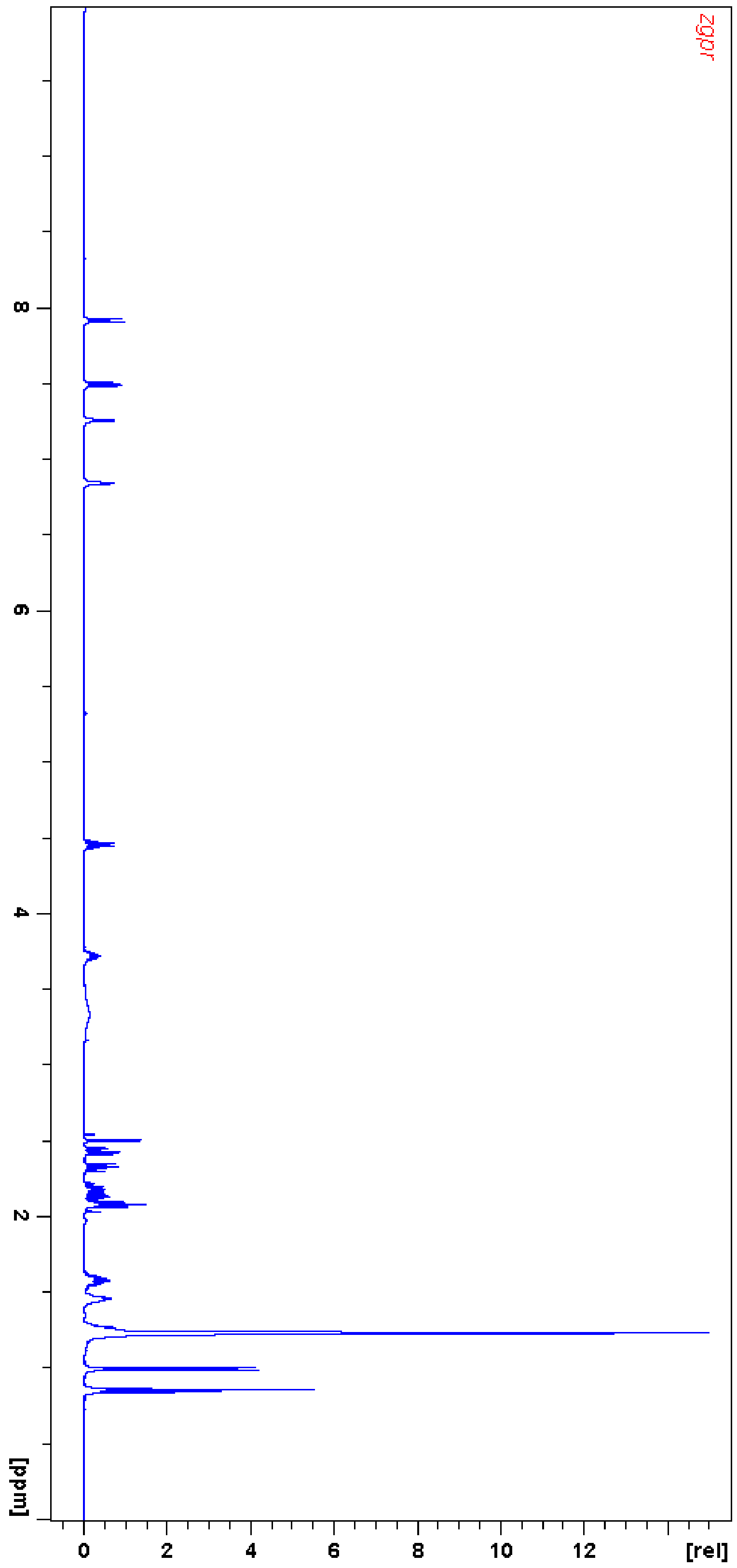

Fig. S3 ^{1}H NMR spectrum of **2** in DMSO-d_6

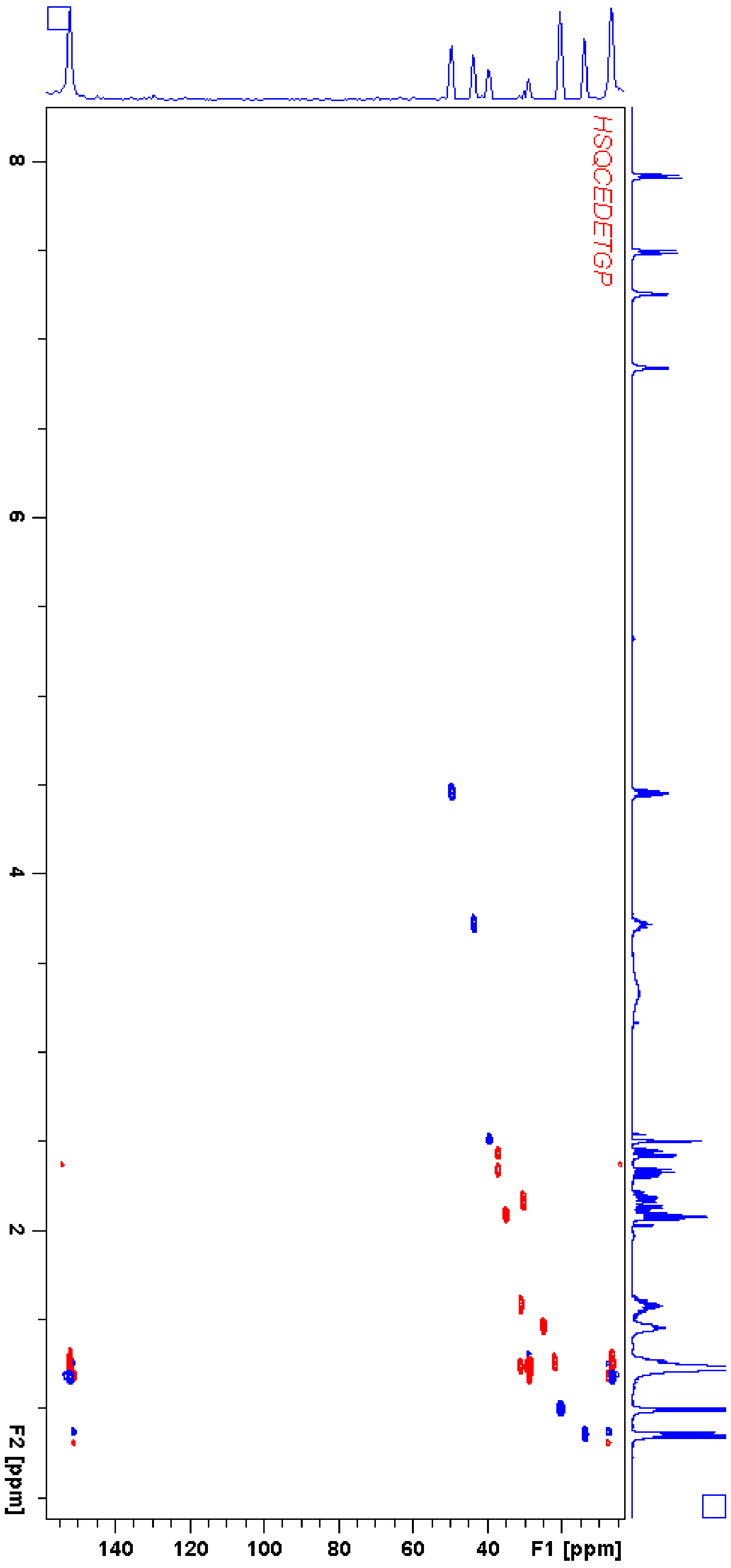

Fig. S4 HSQC spectrum of **2** in DMSO-d_6

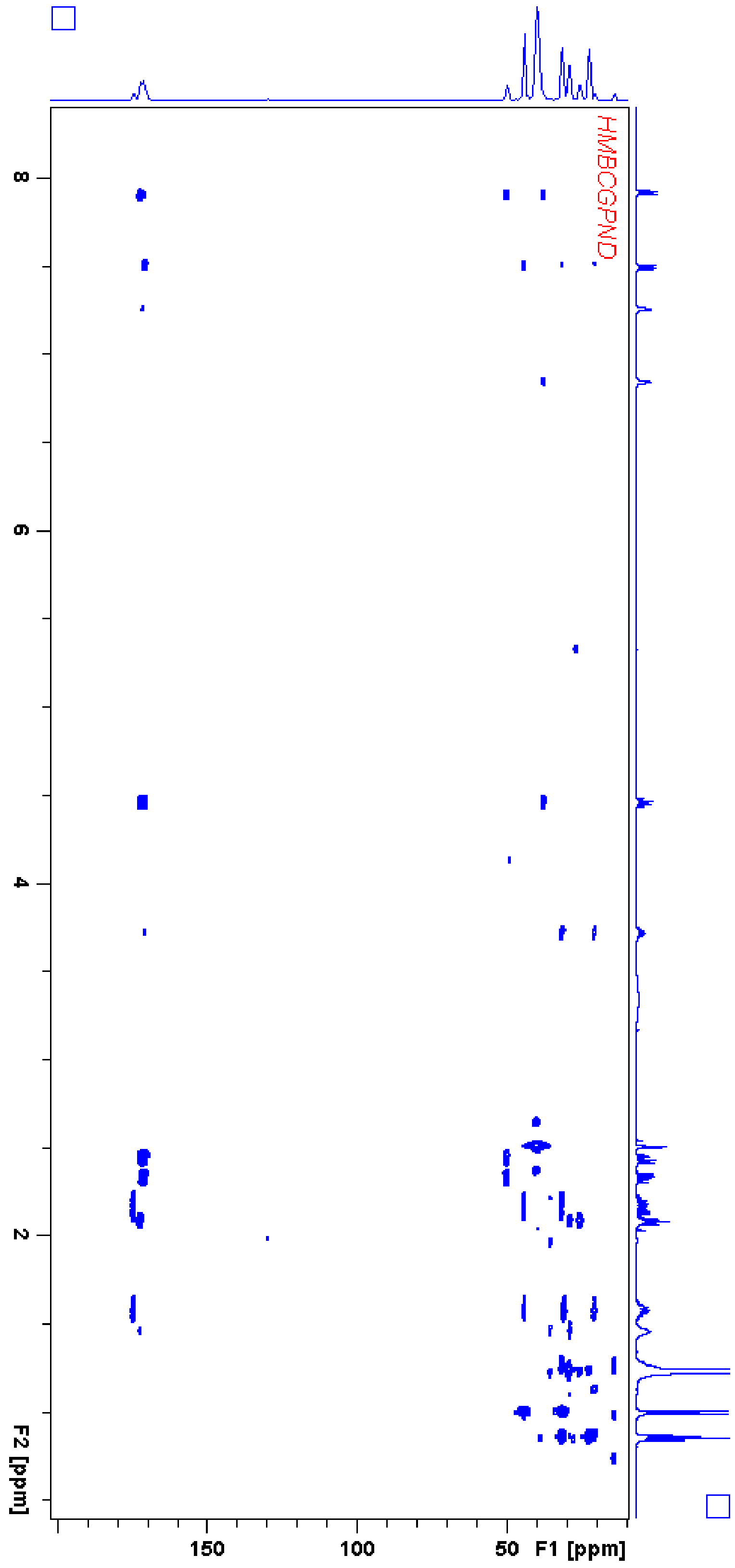

Fig. S5 HMBC spectrum of **2** in DMSO-d_6

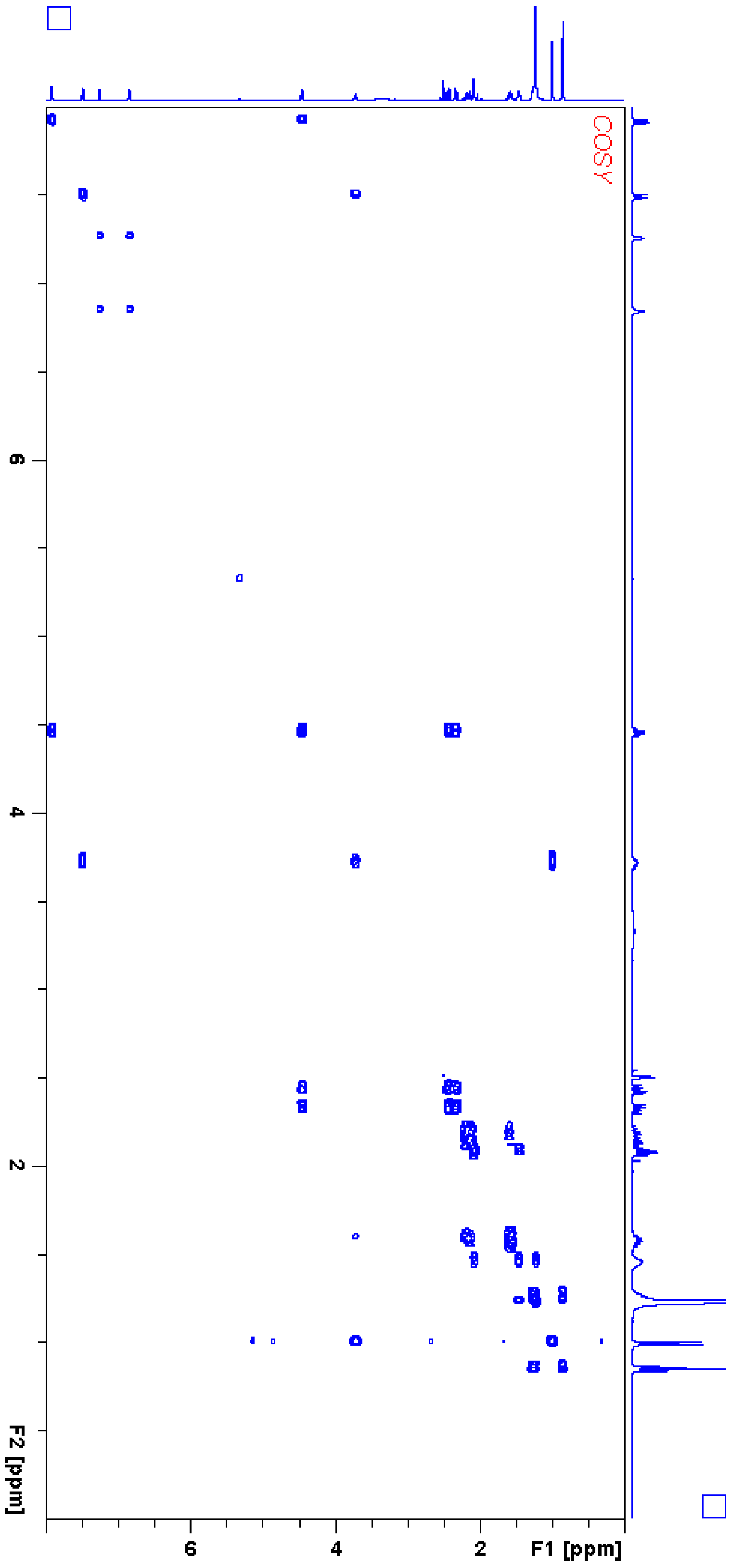

Fig. S6 DQF-COSY spectrum of **2** in DMSO-d_6

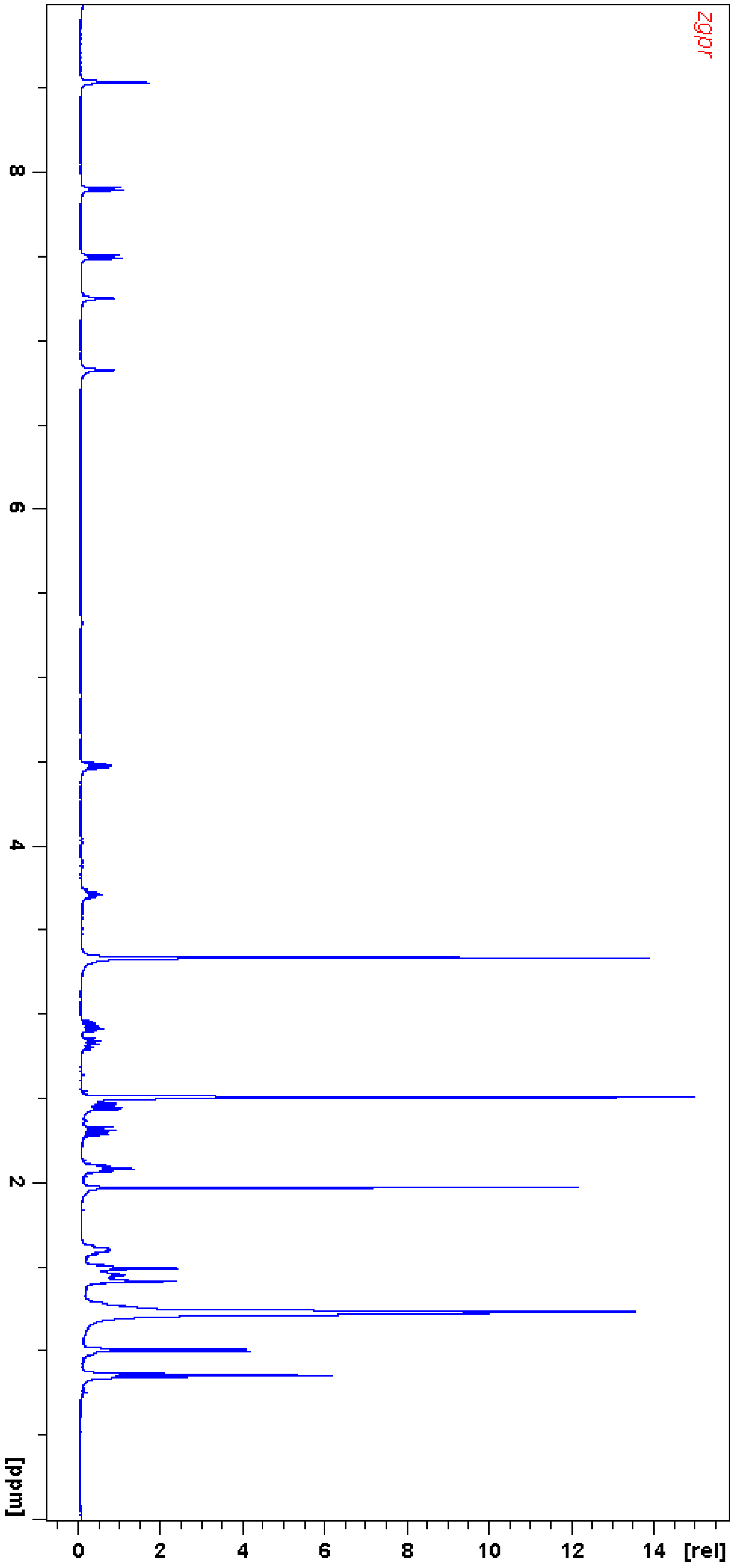

Fig. S7 ^{1}H NMR spectrum of **3** in DMSO-d_6

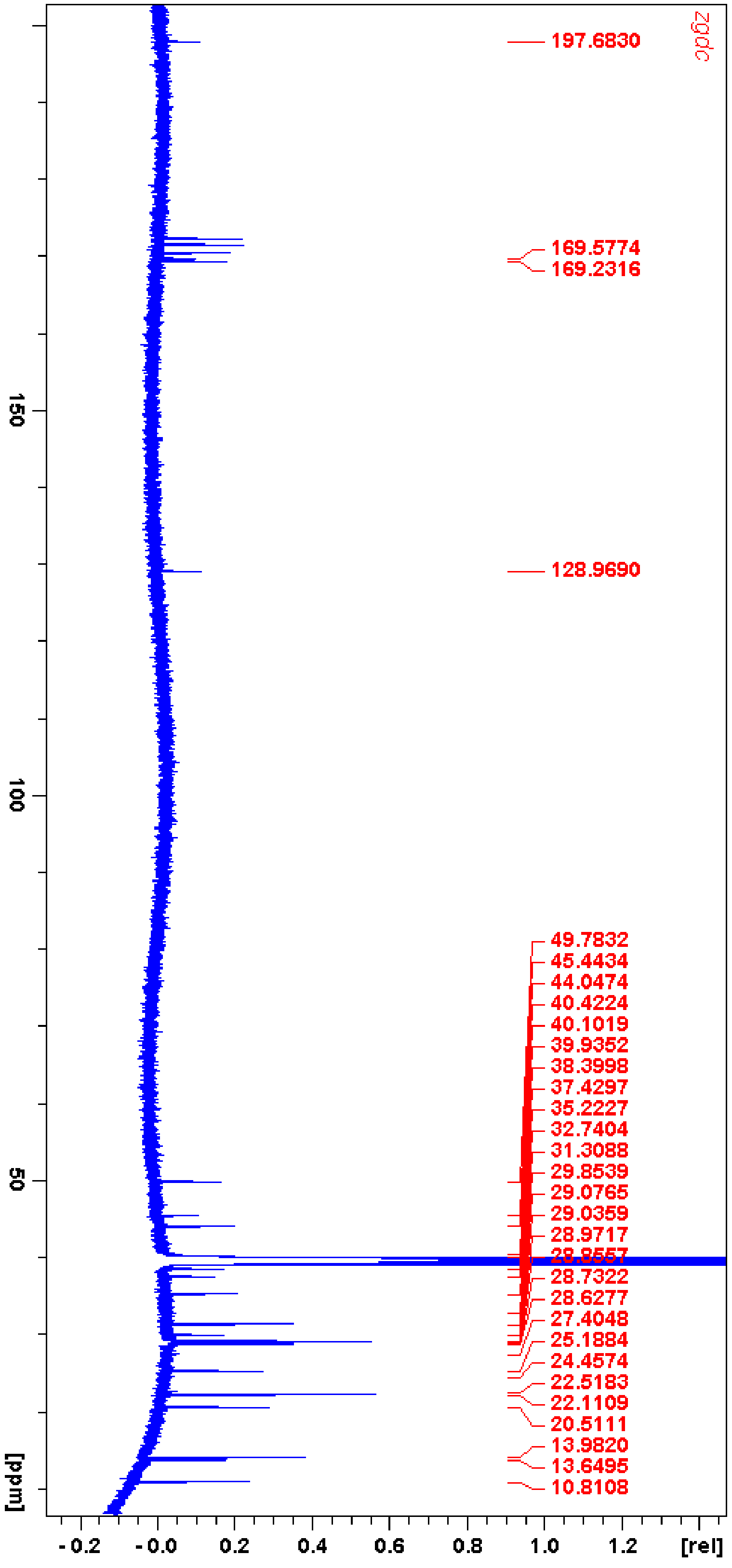

Fig. S8 ^{13}C NMR spectrum of **3** in DMSO-d_6

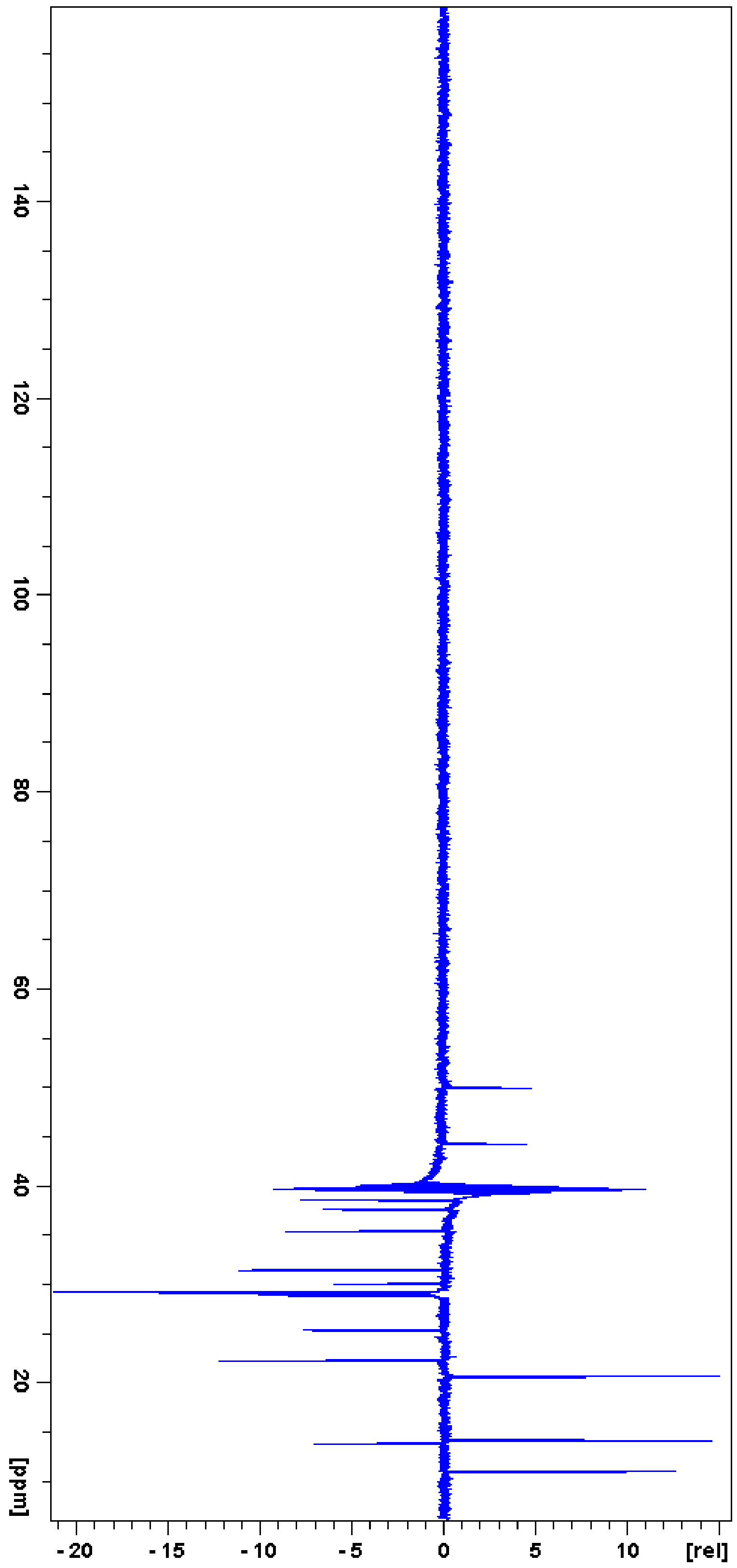

Fig. S9 ^{13}C NMR (DEP-135) spectrum of **3** in DMSO-d_6

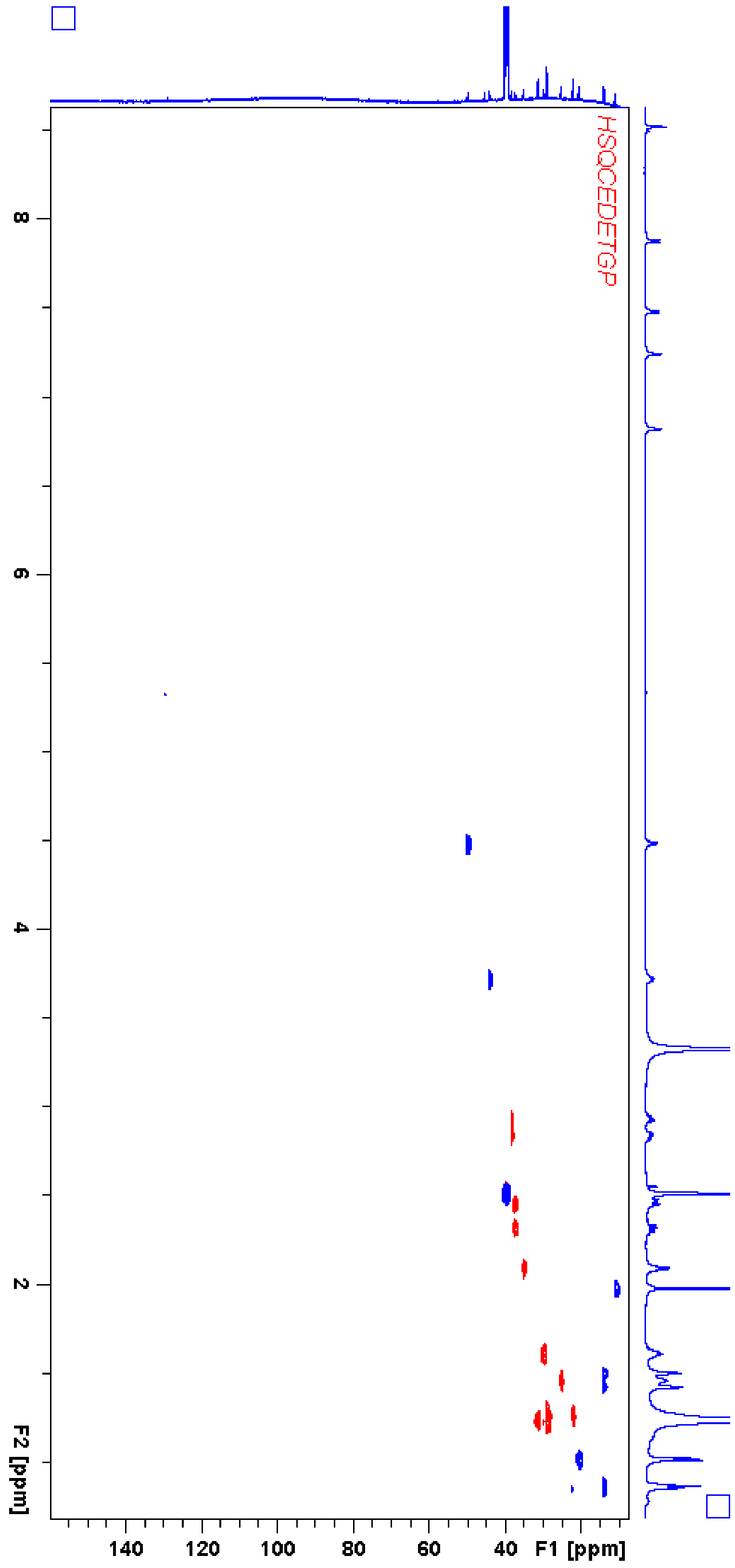

Fig. S10 HSQC spectrum of **3** in DMSO-d_6

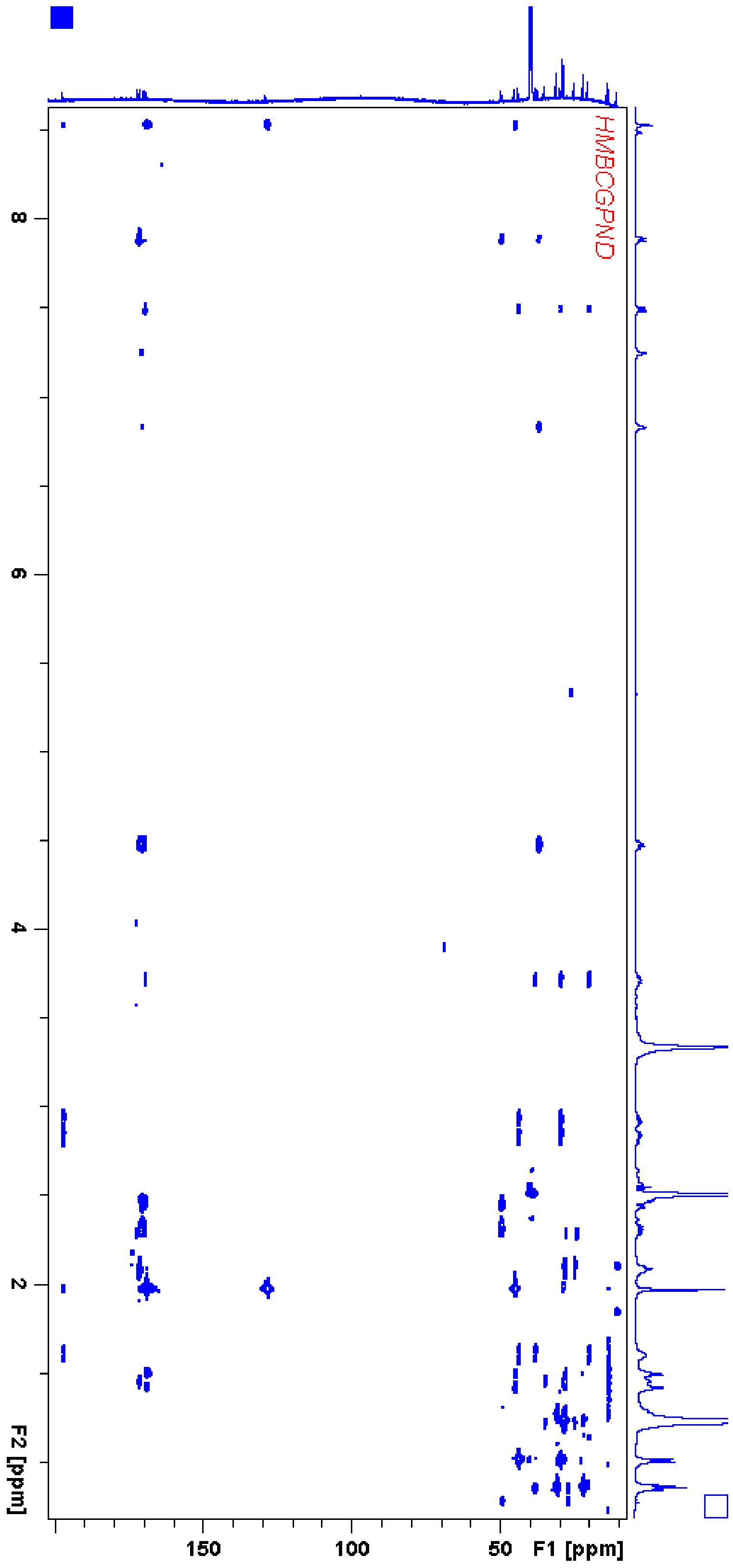

Fig. S11 HMBC spectrum of **3** in DMSO-d_6

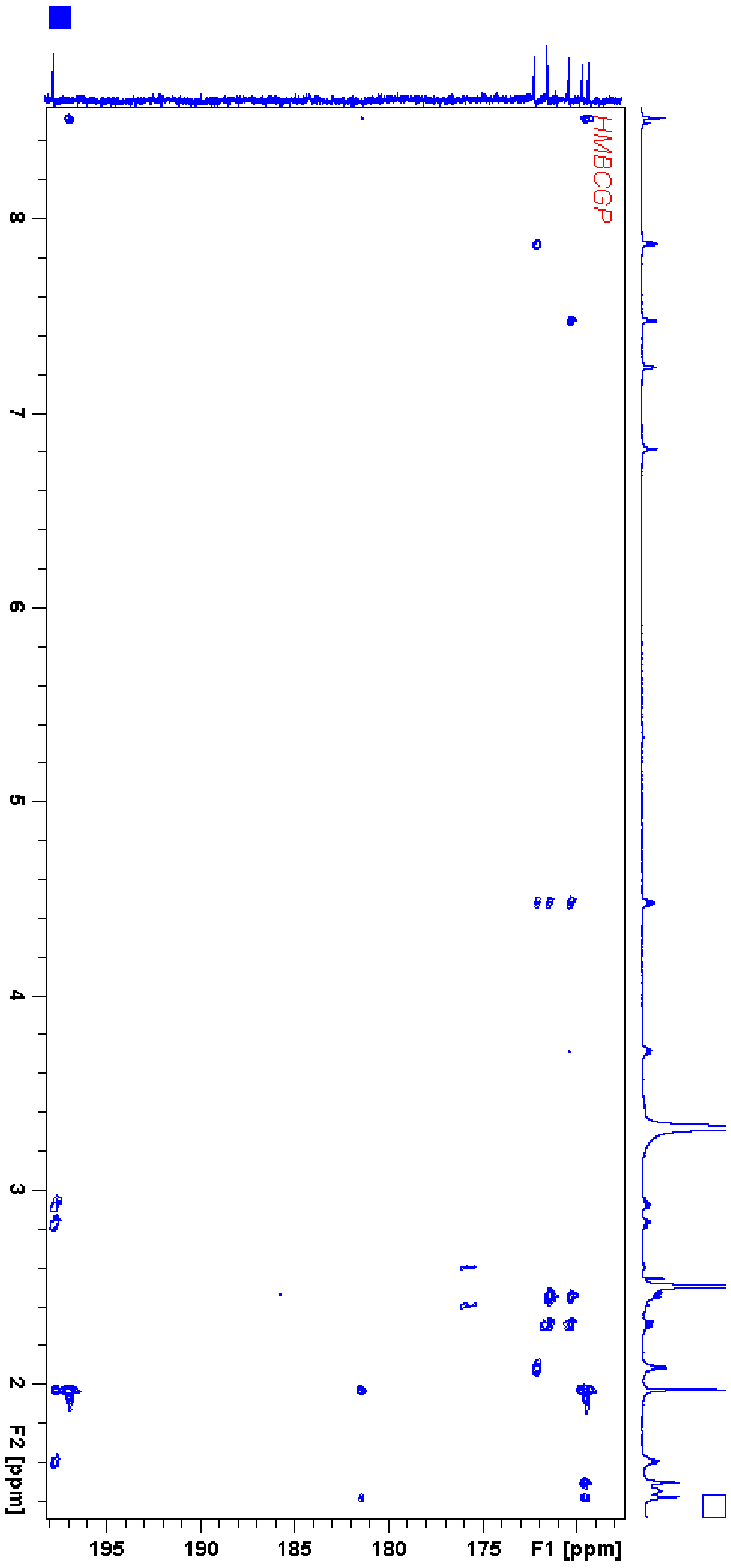

Fig. S12 Selective HMBC spectrum of **3** in DMSO-d_6

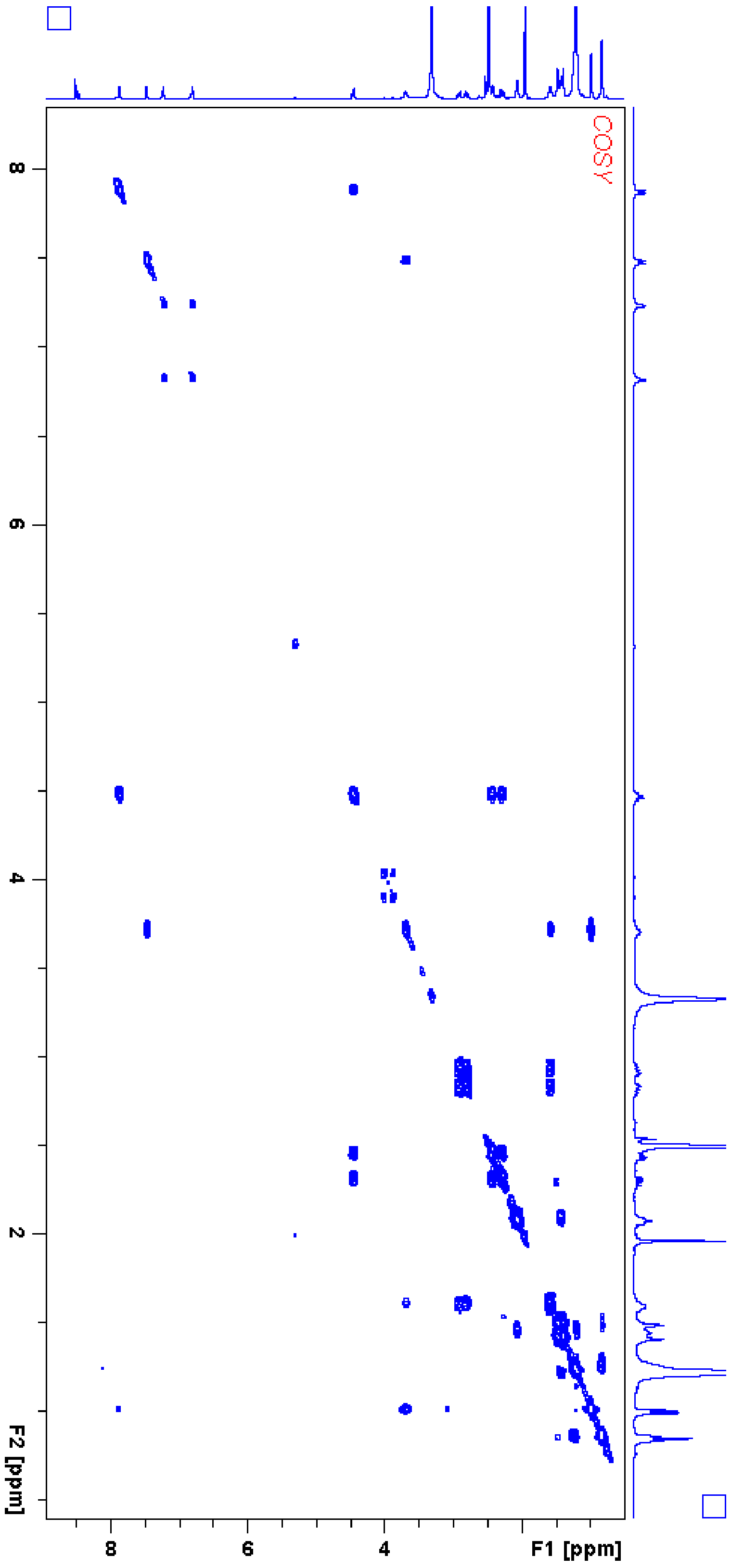

Fig. S13 DQF-COSY spectrum of **3** in DMSO-d_6

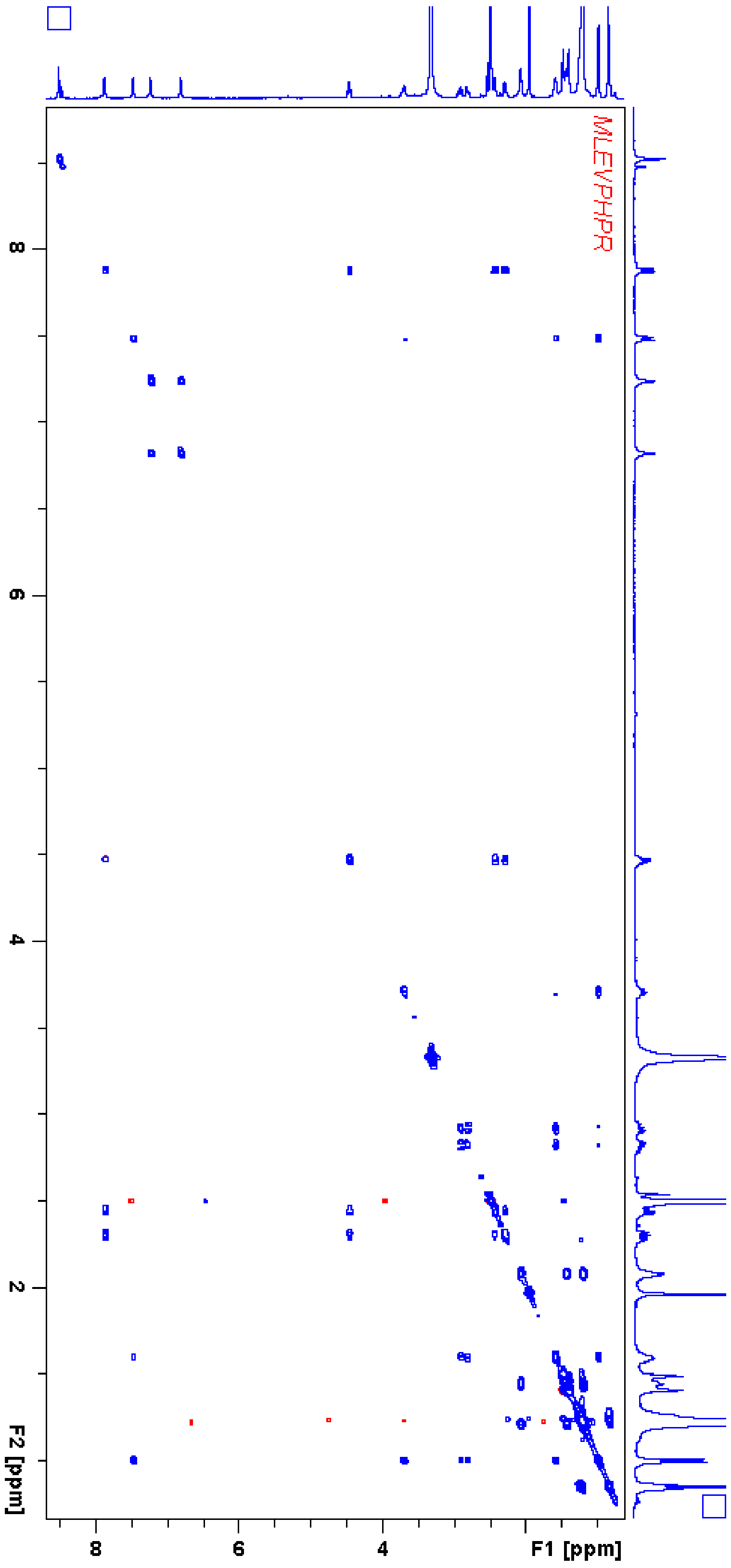

Fig. S14 2D-HOHAHA spectrum of **3** in DMSO-d_6

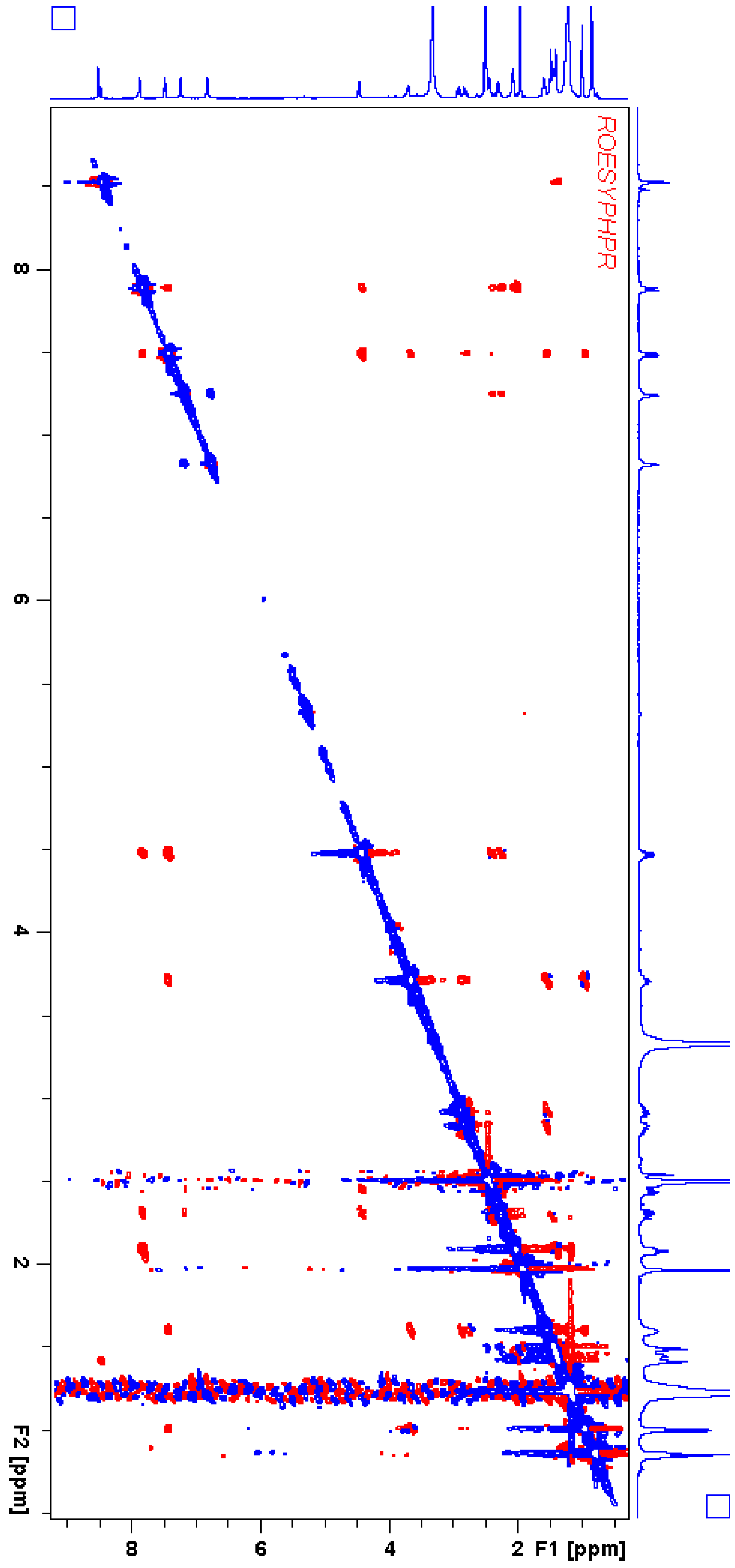

Fig. S15 2D-ROESY spectrum of **3** in DMSO-d_6

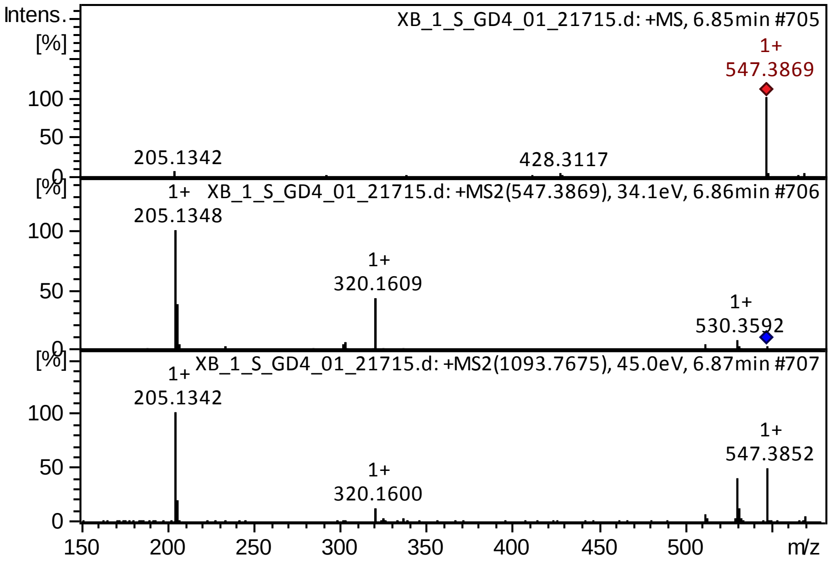

Fig. S16 HR-MS/MS Spectrum (maXis) of compound **3**.

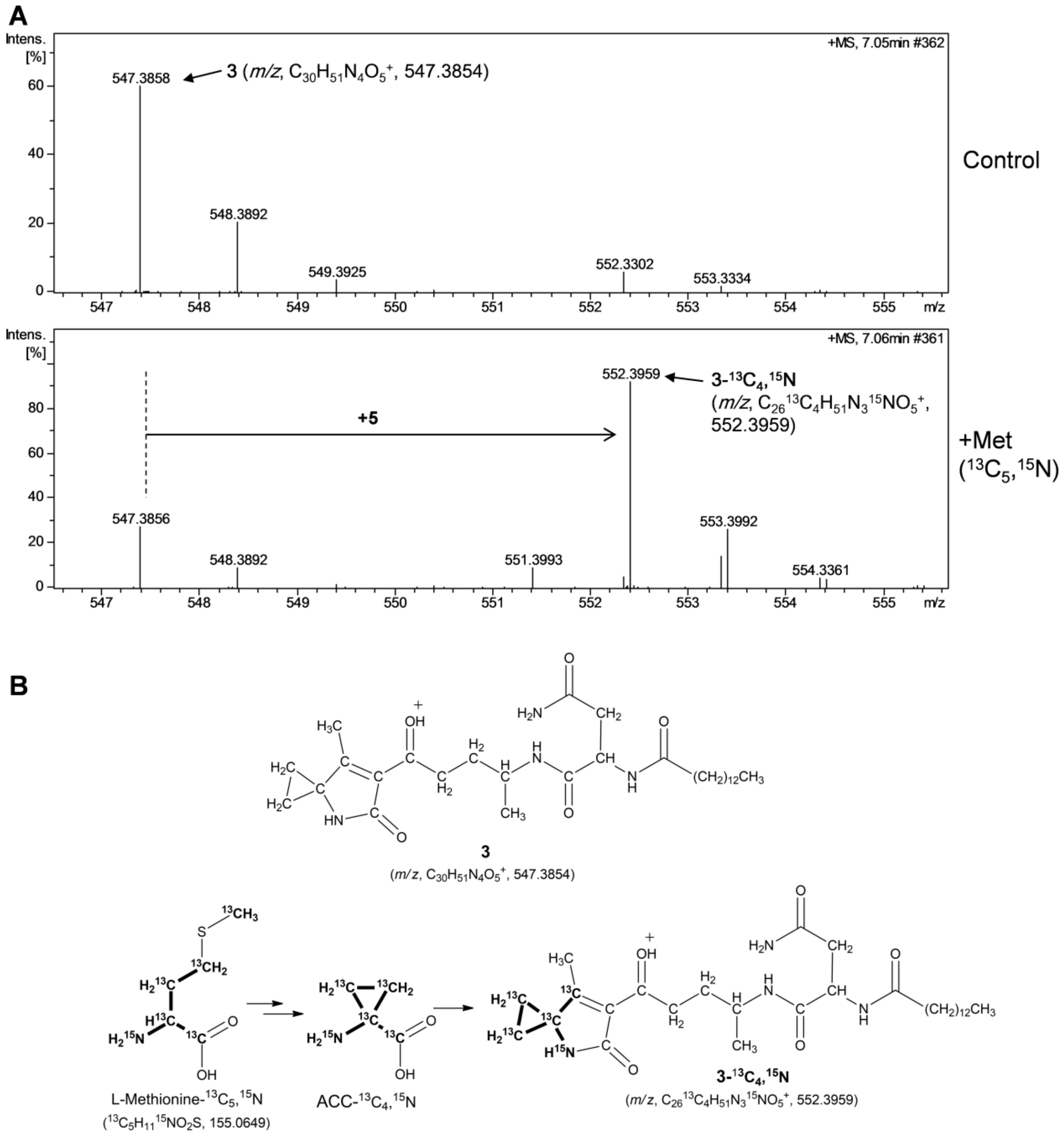

Fig. S17 HRMS analysis of feeding of L-methionine-$^{13}C_5$,^{15}N into *E. coli* Nissle 1917 mutant (A) and their corresponding positions (bold bonds) in compound **3** as well as their theoretical mass-to-charge ratios of labeled compound **3** (B). This feeding experiment indicated that the 1-aminocyclopropanecarboxylic acid (ACC) originates from L -methionine.

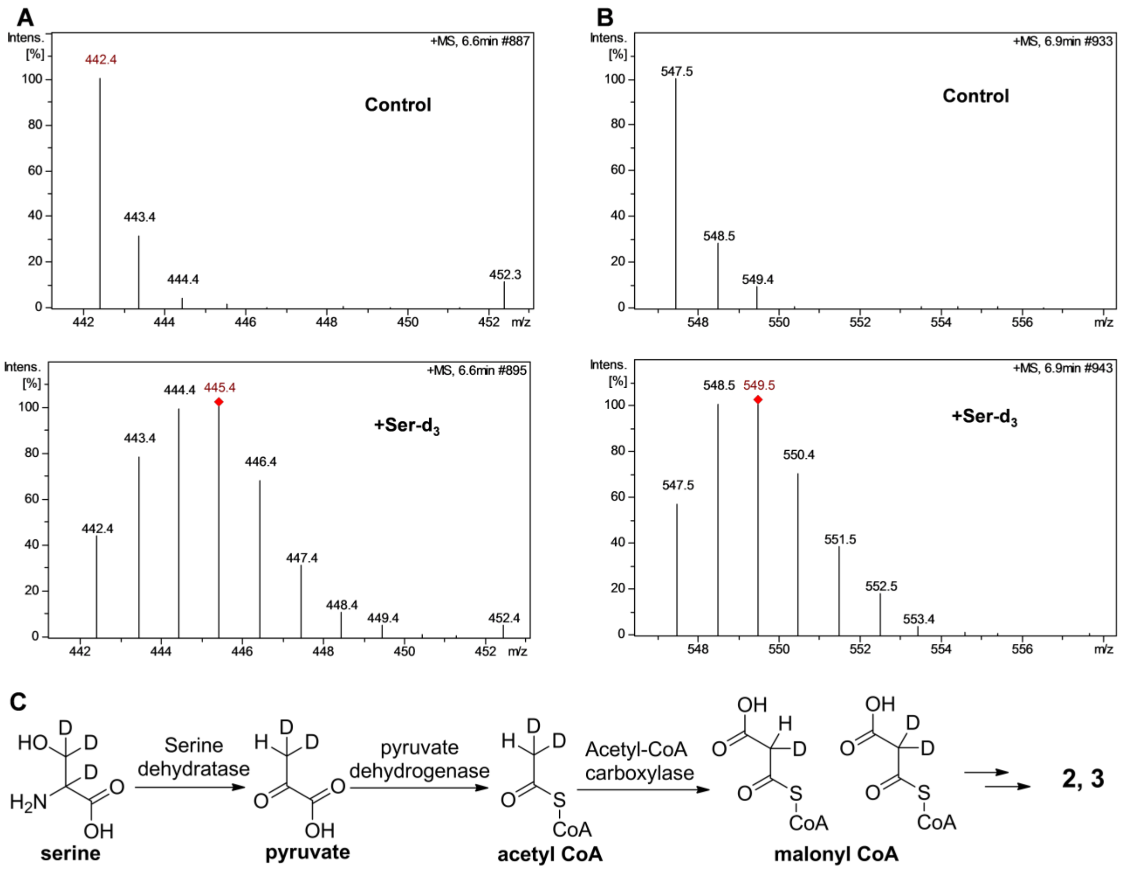

Fig. S18 Feeding of L -serine-d_3 to *E. coli* Nissle 1917 mutant led to multiply deuterated peaks of compounds **2** (A) and **3** (B). The pathway converting serine to malonyl-CoA is also shown (C).

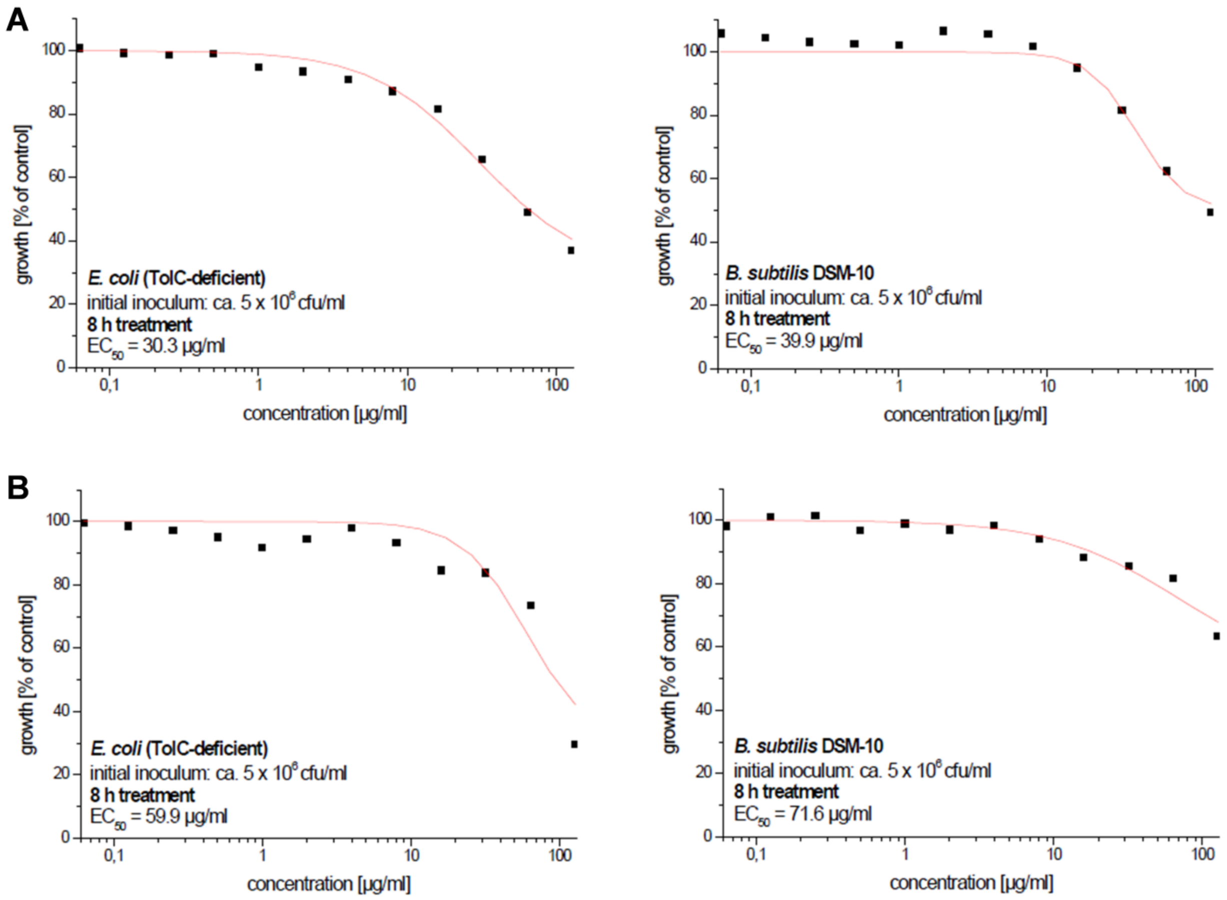

Fig. S19 Growth inhibition of compounds **2** and **3** against *E. coli* (TolC-deficient) and *B. subtillis* DSM-10 after 8h treatment. The EC_{50} are displayed on the graph.

4. Supplementary References

1. X. Bian, J. Fu, A. Plaza, J. Herrmann, D. Pistorius, A. F. Stewart, Y. Zhang, and R. Müller, C*hemBioChem*, 2013, **14**, 1194.
2. J. Fu, S. C. Wenzel, O. Perlova, J. Wang, F. Gross, Z. Tang, Y. Yin, A. F. Stewart, R. Müller, and Y. Zhang, *Nucleic Acids Res.*, 2008, **36**, e113.
3. J. Fu, X. Bian, S. Hu, H. Wang, F. Huang, P. M. Seibert, A. Plaza, L. Xia, R. Müller, A. F. Stewart, and Y. Zhang, *Nat. Biotechnol.*, 2012, **30**, 440.
4. J. Fu, M. Teucher, K. Anastassiadis, W. Skarnes, and A. F. Stewart, *Meth. Enzymol.*, 2010, **477,** 125.
5. J. Wang, M. Sarov, J. Rientjes, J. Fu, H. Hollak, H. Kranz, W. Xie, A. F. Stewart, and Y. Zhang, *Mol. Biotechnol*, 2006, **32,** 43.
6. K. Fujii, Y. Ikai, T. Mayumi, H. Oka, M. Suzuki, and K. Harada, *Anal. Chem.*, 1997, **69**, 3346.
7. K. Fujii, Y. Ikai, H. Oka, M. Suzuki, and K. Harada, *Anal. Chem.*, 1997, **69**, 5146.
8. S. Baumann, J. Herrmann, R. Raju, H. Steinmetz, K. I. Mohr, S. Huttel, K. Harmrolfs, M. Stadler, and R. Müller, *Angew. Chem. Int. Ed.*, 2014, **53**, 14605.
9. F. Surup, K. Viehrig, K. I. Mohr, J. Herrmann, R. Jansen, and R. Müller, *Angew. Chem. Int. Ed.*, 2014, **53**, 13588.
10. B. O. Bachmann and J. Ravel, *Meth. Enymol.*, 2009, **458**, 181.
11. M. Röttig, M. H. Medema, K. Blin, T. Weber, C. Rausch, and O. Kohlbacher, *Nucleic Acids Res., 2*011, **39**, **W**362.
12. C. Prieto, C. Garcia-Estrada, D. Lorenzana, and J. F. Martin, *Bioinformatics, 2*012, **28**, 426.
